# Modulation of Quorum Sensing as an Adaptation to Nodule Cell Infection during Experimental Evolution of Legume Symbionts

**DOI:** 10.1128/mBio.03129-19

**Published:** 2020-01-28

**Authors:** Mingxing Tang, Olivier Bouchez, Stéphane Cruveiller, Catherine Masson-Boivin, Delphine Capela

**Affiliations:** a LIPM, Université de Toulouse, INRAE, CNRS, Castanet-Tolosan, France; b INRAE, US 1426, GeT-PlaGe, Genotoul, Castanet-Tolosan, France; c LABGeM, Génomique Métabolique, Genoscope, Institut François Jacob, CEA, CNRS, Université d’Evry, Université Paris-Saclay, Evry, France; Centro de Ciencias Genómicas, UNAM; University of Texas at Austin

**Keywords:** symbiosis, rhizobium, experimental evolution, infection, quorum sensing, regulation

## Abstract

Over millions of years, changes have occurred in regulatory circuitries in response to genome reorganization and/or persistent changes in environmental conditions. How bacteria optimize regulatory circuitries is crucial to understand bacterial adaptation. Here, we analyzed the experimental evolution of the plant pathogen Ralstonia solanacearum into legume symbionts after the transfer of a natural plasmid encoding the essential mutualistic genes. We showed that the Phc quorum sensing system required for the virulence of the ancestral bacterium was reconfigured to improve intracellular infection of root nodules induced by evolved *Ralstonia*. A single mutation in either the PhcB autoinducer synthase or the PhcQ regulator of the sensory cascade tuned the kinetics of activation of the central regulator PhcA in response to cell density so that the minimal stimulatory concentration of autoinducers needed for a given response was increased. Yet, a change in the expression of a PhcA target gene was observed in infection threads progressing in root hairs, suggesting early programming for the late accommodation of bacteria in nodule cells. Moreover, this delayed switch to the quorum sensing mode decreased the pathogenicity of the ancestral strain, illustrating the functional plasticity of regulatory systems and showing how a small modulation in signal response can produce drastic changes in bacterial lifestyle.

## Abstract (precis)

Rhizobia are soil bacteria from unrelated genera able to form a mutualistic relationship with legumes. Bacteria induce the formation of root nodules, invade nodule cells, and fix nitrogen to the benefit of the plant. Rhizobial lineages emerged from the horizontal transfer of essential symbiotic genes followed by genome remodeling to activate and/or optimize the acquired symbiotic potential. This evolutionary scenario was replayed in a laboratory evolution experiment in which the plant pathogen Ralstonia solanacearum successively evolved the capacities to nodulate Mimosa pudica and poorly invade, then massively invade, nodule cells. In some lines, the improvement of intracellular infection was achieved by mutations modulating a quorum sensing regulatory system of the ancestral strain. This modulation that affects the activity of a central regulator during the earliest stages of symbiosis has a huge impact on late stages of symbiosis. This work showed that regulatory rewiring is the main driver of this pathogeny-symbiosis transition.

## INTRODUCTION

Most bacterial cellular functions are regulated by complex multilayered genetic regulatory networks that include environmental and internal sensors and a wide variety of signaling pathways. Fine-tuned regulation allows rapid shifts in metabolism, physiology, and behavior in response to environmental fluctuations (1–3). Over millions of years, the regulation circuits have evolved to adapt to genome reorganization and/or persistent changes in environmental conditions (4). Deciphering how bacteria optimize regulatory circuitries is crucial to understand bacterial adaptation and the biosphere.

Quorum sensing (QS) systems are key components of regulatory networks in bacteria which allow the regulation of gene expression in a population-dependent manner. QS involves the production of extracellular signals, called autoinducers, whose concentration is a function of cell population density. Above a system-specific threshold level, these molecules rapidly activate or repress the transcription of hundreds of genes, thus modifying and synchronizing bacterial behavior on a population-wide scale. These signal response systems are widespread in both Gram-positive and Gram-negative bacteria, where extensive variations regarding the type of autoinducers and cognate receptors, signal transduction mechanisms, and associated cellular responses have been described (5). Biological functions regulated by QS are numerous, including bioluminescence, virulence, symbiosis, motility, biofilm formation, or exopolysaccharide (EPS) production (6–10). The plant pathogen Ralstonia solanacearum possesses a virulence-related QS system, the Phc system, which controls the activity of the LysR-type transcription regulator PhcA (11). One component of this system is PhcB, a protein harboring methyltransferase activity essential for the synthesis of autoinducers, either (*R*)-3-hydroxypalmitic acid methyl ester [(*R*)-3-OH PAME] or (*R*)-3-hydroxymyristic acid methyl ester [(*R*)-3-OH MAME], depending on the R. solanacearum strains. In strain GMI1000, only (*R*)-3-OH MAME was detected (12). How this signal is transduced to PhcA has not been fully elucidated. At low cell density, the unphosphorylated two-component system PhcSR is thought to inhibit the activity of the central virulence regulator PhcA via an unknown mechanism. When the QS molecules reach the minimal stimulatory concentration (around 10 nM, matching the cell density of 10^7^ CFU/ml in liquid culture), they likely trigger the autophosphorylation of the sensor histidine kinase PhcS, which, in turn, phosphorylates the response regulator PhcR (13), and this releases the inhibition of PhcA (14). The *phcQ* gene, which is likely cotranscribed with *phcBSR*, encodes a protein composed of a receiver domain similar to the receiver domain of PhcR (see Fig. S1A in the supplemental material). The role of this protein in the QS-dependent signaling cascade is not known (14). Activation of PhcA in R. solanacearum allows the switch from metabolic versatile cells to nonmotile EPS-producing virulent cells (15). Three independent transcriptomic studies conducted under three different experimental conditions (minimal medium, rich medium, and *in planta*) indeed identified hundreds of genes under the positive or negative control of PhcA (16–18).

10.1128/mBio.03129-19.1 FIG S1 (A) Representation of the R. solanacearum *phcBSRQ* operon and structural domains of the corresponding proteins. SAMdM, *S*-adenosyl-l-methionine-dependent methyltransferase; TMD, transmembrane domains; D/PAD. dimerization/phosphoacceptor domain; HK. histidine kinase; RD, receiver domain. Arrows indicate the mutated positions in *phcB* (R22C), *phcS* (L161R), and *phcQ* (R154C). (B) Prediction of the 3-dimensional structures of the wild-type and mutated PhcB and PhcQ proteins. Structures were predicted using the Phyre2 server (L. A. Kelley, S. Mezulis, C. M. Yates, M. N. Wass, and M. J. Sternberg, Nat Protoc 10:845–858, 2015, https://www.doi.org/10.1038/nprot.2015.053). (C) Protein alignments showing the conservation of the R22 and R154 amino acids of the PhcB and PhcQ proteins, respectively. The amino acid sequences of PhcB/Q from R. solanacearum GMI1000 were blasted against the nonredundant NCBI database to obtain a list of homologs from other organisms with identity ranging from 64% to 100% for PhcB and 53% to 100% for PhcQ. A subset of hits, covering the diversity of genera and species containing *phcB* and *phcQ* homologs, is presented. Sequences were aligned using MultAlin (http://multalin.toulouse.inra.fr/multalin/multalin.html). Red, amino acid residues highly conserved (>90%); black, positions neutrally conserved (50% to 90%); blue, positions lowly conserved (<50%). Download FIG S1, PDF file, 0.6 MB. Copyright © 2020 Tang et al. 2020 Tang et al. This content is distributed under the terms of the Creative Commons Attribution 4.0 International license.

Rhizobia, the nitrogen-fixing symbionts of legumes, are an excellent biological system to investigate how regulatory circuits rewire in response to genome reorganization and colonization of new niches. Rhizobia are soil bacteria able to induce the formation of root nodules where internalized bacteria fix nitrogen for the benefit of the plant (19). Extant rhizobia belong to hundreds of species distributed in 18 different genera of alpha- and betaproteobacteria (20–22). They have evolved through the horizontal acquisition of essential symbiotic genes, the nodulation (*nod*) and nitrogen fixing (*nif-fix*) genes, likely followed by subsequent adaptation of the recipient genome to the new legume endospheric environment (20). To understand posthorizontal gene transfer adaptation, we previously experimentally evolved Ralstonia solanacearum into legume symbionts by mimicking the natural evolutionary scenario. The symbiotic plasmid pRalta of the rhizobium Cupriavidus taiwanensis LMG19424 harboring the *nod* and *nif-fix* genes (23) was transferred to R. solanacearum strain GMI1000, and the resulting chimera was then evolved in parallel lineages using serial plant-bacteria cocultures. While the ancestral strain was strictly extracellular and unable to form root nodules, most lines sequentially acquired the capacity to form root nodules, poorly infect nodules, and massively infect nodule cells (24, 25). Acquisition of nodulation relied on the inactivation of the R. solanacearum pathogenic type III secretion system (T3SS) (24). The first level of infection was gained via inactivation of virulence regulators, either HrpG or VsrA (24, 26). In some lines, infection was optimized via modifications in the EfpR regulatory pathway (27). EfpR was shown to be a global virulence regulator and a metabolic repressor (27, 28). In other very infectious lines, no mutation was detected in known components of the EfpR pathway, suggesting that evolution targeted either unknown components of this path or another pathway.

Here, we show that in some of these lines, massive intracellular accommodation was reached via modifications in the R. solanacearum quorum sensing system. Mutations in either *phcB* or *phcQ* allowed improved infection via a fine modulation of the sensory pathway that delays to different degrees the cell density-dependent activation of the central regulator PhcA. While the highly similar gene expression profiles of *efpR* and *phcA* mutants suggested a connection between the two pathways, present results indicated that EfpR interferes with the PhcA regulon independently from the PhcBSRQ sensory cascade. Both *efpR* and *phc* late infection-adaptive mutations transiently affect the expression of PhcA target genes during early symbiotic stages corresponding to the entry and progression of bacteria in root hairs. EfpR and PhcA may thus control the expression of a common set of genes whose deregulation helps change the lifestyle of the bacterium, improving its symbiotic infection capacity while decreasing its pathogenicity.

## RESULTS

### Infection-adaptive mutations affect the *phc* system in E, K, and M lineages.

**FIG 1:**
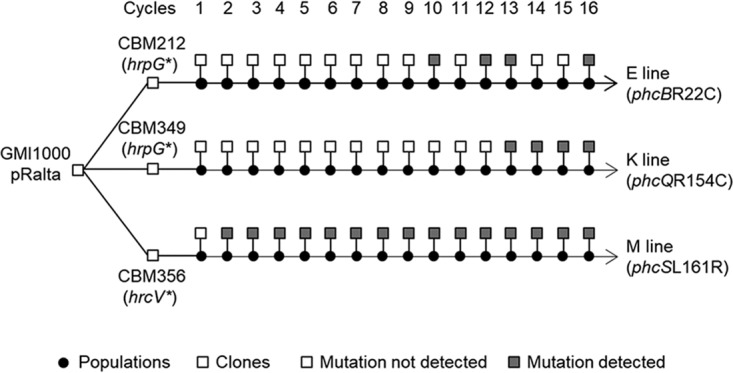
E, K, and M lineages of the experimental evolution of R. solanacearum GMI1000. The E, K, and M lines were independently derived from the spontaneous nodulating mutants of GMI1000(pRalta) CBM212, CBM349, and CBM356, respectively. Sixteen serial cycles of inoculation to *M. pudica-*isolation of nodule bacteria were performed for each lineage (30). At each cycle, a sample of the nodule bacterial population was inoculated to new plants and the rest of the population stored at −80°C. Populations were serially diluted, and one representative clone was randomly selected from the highest dilution plate for further phenotypic and genomic analyses. Bacteria of these lines carry a stop mutation in either *hrpG* or *hrcV* (24), depending on the ancestor, which provides strains the capacity to nodulate *M. pudica*. The *phcB*R22C, *phcQ*R154C, and *phcS*L161R mutations were identified in clones isolated from the E, K, and M lines, respectively, by either Illumina genome resequencing, PCR amplification and Sanger sequencing, or PCR screening of wild-type and mutated alleles (see Text S1 in the supplemental material).

Our previous work showed that the evolved clones E16, K16, and M16 bore adaptive mutations in genes encoding components of the Ralstonia solanacearum Phc quorum sensing system. Nonsynonymous mutations in the *phcB* (*phcB*R22C), *phcQ* (*phcQ*R154C), and *phcS* (*phcS*L161R) genes (Table 1) improved the *in planta* fitness (evaluated by the number of bacteria in nodule populations) of the evolved clones compared to that of the derivatives harboring the wild-type alleles (29). Genome resequencing or PCR analysis of intermediate clones along the E, K, and M lineages allowed the detection of the *phcB*R22C mutation in half clones from cycle 10 in the E lineage and the detection of *phcQ*R154C and *phcS*L161R mutations in all clones from cycles 13 and 2 in the K and M lineages, respectively (Fig. 1). This suggested that these mutations have fixed rapidly in the K and M lineages and less rapidly in the E lineage.

**TABLE 1:** Validated infection-adaptive mutations in E, K, and M lineages

Strain(s)	Gene	Product	Mutation	Position on the chromosome	Protein modification
E16	RSc2735, *phcB*	Regulatory protein, SAM-dependent methyltransferase domain	C/T	2943477	R22C
K16	RSc2738, *phcQ*	Response regulator receiver, CheY family	C/T	2947813	R154C
M5, M16	RSc2736, *phcS*	Sensor protein histidine kinase, repressor of PhcA	T/G	2945312	L161R

10.1128/mBio.03129-19.9 TEXT S1 Supplemental materials and methods. Download Text S1, DOCX file, 0.1 MB. Copyright © 2020 Tang et al. 2020 Tang et al. This content is distributed under the terms of the Creative Commons Attribution 4.0 International license.

**FIG 2:**
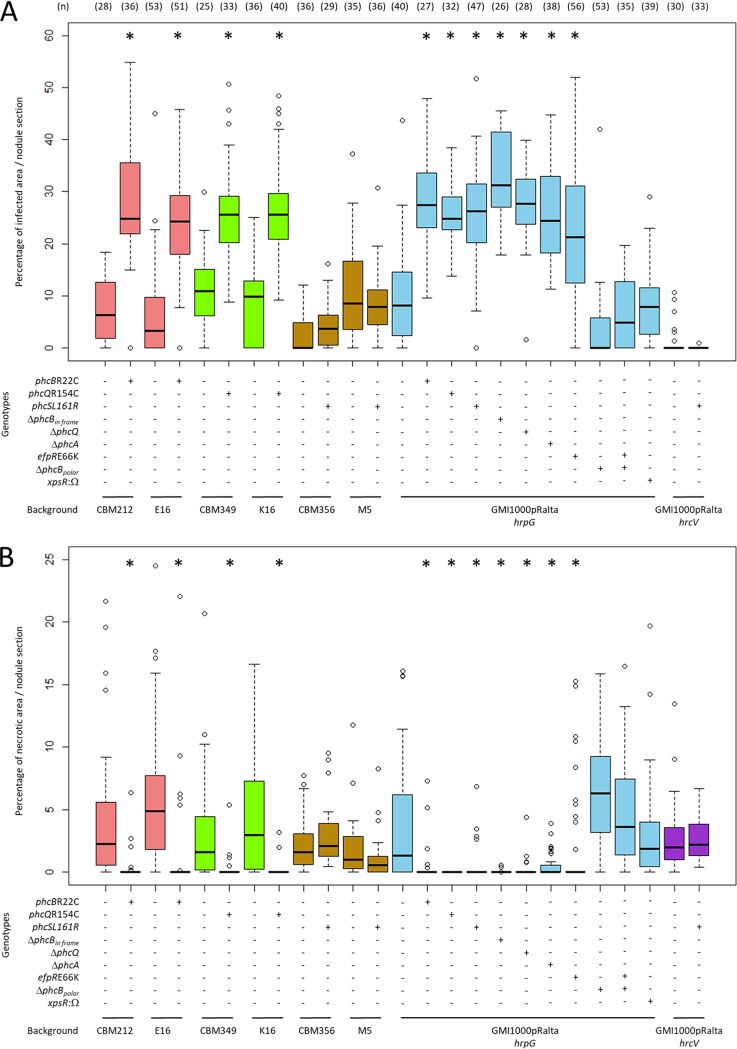
Quantification of the relative infected and necrotic areas of nodules induced by evolved clones and mutants. Distributions of the percentages of infected areas (A) and necrotic areas (B) per nodule section recovered at 21 dpi. The central rectangles span the first quartiles to the third quartiles (that is, the interquartile range [IQR]), the bold segments inside the rectangles show the medians, unfilled circles indicate outliers, whiskers above and below the boxes show either the locations of the minimum and maximum in the absence of suspected outlying data or 1.5 IQR if an outlier is present. Δ*phcB*_in frame_, nonpolar deletion of *phcB*. Data for *efpR*E66K are from reference 25. *, *P < *0.05 versus the respective ancestral strain, either CBM212, CBM349, CBM356, or GMI1000(pRalta) *hrpG* (multiple-comparison test after Kruskal-Wallis). Nodules were obtained from at least two independent experiments. For each experiment, nodules were harvested from at least 16 plants. *n*, number of nodules analyzed.

To further determine whether these mutations improve nodule cell infection, we inoculated Mimosa pudica individually with E16, K16, M5, their derivatives harboring the wild-type (WT) *phc* alleles, their respective nodulating ancestors CBM212, CBM349, and CBM356, and mutants of these ancestors carrying the *phc* mutations (M5, which is statistically as infectious as M16 [26], was used rather than M16, since genetic transformation failed in the latter clone in spite of many trials). We measured the size of the intracellularly infected and necrotic zones, identified as light-brown and dark-brown zones, respectively (25, 30), in sections of nodules collected at 21 days postinoculation (dpi). These two parameters were used as indicators of the quantity and quality of infection. The intracellular infection capacity of E16, CBM212 *phcB*R22C, K16, and CBM349 *phcQ*R154C increased significantly compared to that of E16 *phcB*WT, CBM212, K16 *phcQ*WT, and CBM349, respectively (Fig. 2A). At the same time, the capacity to induce necrosis decreased in the strains bearing the mutated alleles (Fig. 2B). Reconstructing the *phcB* and *phcQ* mutations in the chimeric *Ralstonia* strain GMI1000(pRalta) harboring an *hrpG* mutation (CBM1627), conferring nodulation and partial infection ability, provided this strain a capacity to infect nodule cells and induce necrosis similarly to that of the evolved E16 or K16 clones, respectively, indicating that these mutations are the major infection-adaptive ones in E and K lines (Fig. 2).

The effect of the *phcS*L161R mutation depended on the genetic background (Fig. 2). It improved intracellular infection and reduced necrosis in GMI1000(pRalta) *hrpG* but failed to do so in *hrcV*-mutated backgrounds [M5, CBM356, and GMI1000(pRalta) *hrcV*]. This result is not necessarily contradictory to the previously observed increase in relative *in planta* fitness of CBM356 *phcS*L161R and M5 compared to that of CBM356 and M5 *phcS*WT (29), which could be due to an increase in nodulation competitiveness or extracellular infection rather than an increase in intracellular infection *per se*.

The reconstruction of the *phcBQS* mutations in GMI1000(pRalta) did not allow the original chimeric strain to nodulate *M. pudica*, indicating that the effect of these mutations is conditional on the presence of the *hrpG* stop mutation.

Altogether, this showed that the *phcB*R22C and *phcQ*R154C, but not *phcS*L161R, were the main infection-enhancing mutations in their respective lineages. We thus further analyzed the effect of the *phcB* and *phcQ* mutations on the Phc quorum sensing system.

### Infection-adaptive *phcBQ* mutations do not constitutively inactivate the PhcA transcription regulator.

The cell density-responsive regulatory system PhcBSRQ controls the PhcA central regulator (11). To evaluate whether the infection-adaptive *phc* mutations led to the inactivation of the Phc quorum sensing system, we evaluated the activity of PhcA in wild-type and mutated strains. For that, we constructed a plasmidic fusion between the promoter region of *xpsR*, a direct transcriptional target of PhcA (31), and the Escherichia coli reporter gene *lacZ* encoding β-galactosidase. We introduced this plasmid in GMI1000(pRalta) *hrpG*, in its *phcB*R22C, *phcQ*R154C single nucleotide polymorphism (SNP) derivative mutants, and in its *phcB-* (in frame), *phcQ-*, and *phcA*-deleted mutants. Since *xpsR* expression is under positive control of PhcA, the dosage of β-galactosidase activity positively correlates with PhcA activity.

**FIG 3:**
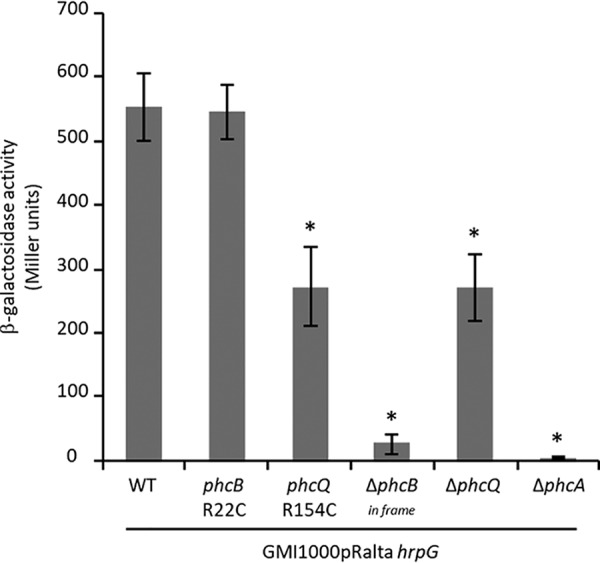
Expression of the p*xpsR-lacZ* plasmidic fusion in the chimeric R. solanacearum nodulating strain and derivative mutants at mid-exponential phase. β-Galactosidase activities were measured on bacteria grown to mid-exponential phase (OD_600_ was around 0.2) in rich BG medium. Data are from 3 independent experiments. Values correspond to means ± standard deviations. Δ*phcB*_in frame_, nonpolar deletion of *phcB*. *, *P* < 0.05; **, *P* < 0.001 versus the wild-type strain (Student’s *t* test).

The β-galactosidase activity was determined from mid-exponentially grown liquid cultures of each strain in rich medium. In agreement with the literature (31), deletion of *phcA* completely abolished *xpsR-lacZ* expression (Fig. 3). In contrast, the expression of *xpsR-lacZ* in the *phcB* SNP mutants was high and similar to that of strain GMI1000(pRalta) *hrpG*, and the *phcQ* SNP mutant only exhibited a lower level of reporter gene expression (Fig. 3). The *phcB* SNP mutant was clearly different from a *phcB* in-frame deletion mutant, indicating that the mutation did not inactivate the protein. In contrast, the *phcQ* deletion mutant exhibited a similar level of *xpsR-lacZ* reporter gene expression as the *phcQ*R154C mutant.

**FIG 4:**
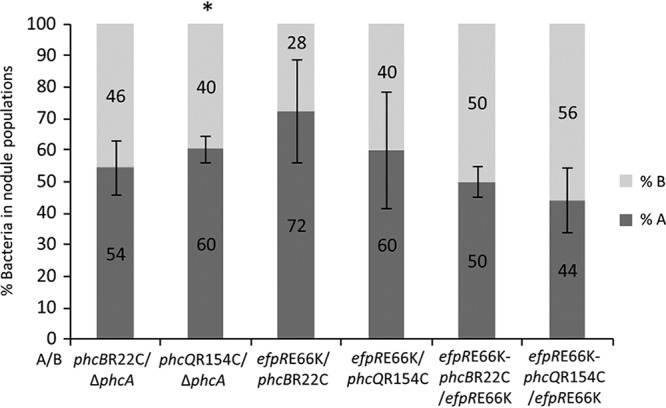
Relative *in planta* fitness of infection-adaptive mutants. *Mimosa pudica* plantlets were coinoculated with pairs of mutants at a 1:1 ratio. Nodules were harvested at 21 dpi, and the number of bacteria from each strain in the bacterial nodule population was measured by plating dilutions of nodule crushes and using either PCR for detecting the presence/absence of mutations in colonies or fluorescent markers as screening methods. The percentages of bacteria in nodule populations were normalized by the inoculum ratio. Values correspond to means ± standard deviations. *, *P* < 0.05 between strains (Student’s *t* test).

Surprisingly, the three Δ*phcA*, Δ*phcB_in frame_*, and Δ*phcQ* mutants induced on *M. pudica* the formation of nodules with infection and necrosis zones similar to that of nodules induced by the *phcBQ* SNP mutants (around 25% to 30% of the nodule section) (Fig. 2). Moreover, the *phcB* and *phcQ* SNP mutants display either no or a minor difference in relative *in planta* fitness from that of a Δ*phcA* mutant, indicating that a *phcA* deletion is almost as adaptive as the *phcB*R22C and *phcQ*R154C mutations for symbiosis (Fig. 4). This suggested that the point mutations in *phcB* and *phcQ* affect respective protein functions, resulting in an altered PhcA activity in symbiosis.

### Infection-adaptive *phc* mutations decrease responsiveness to cell density.

The *phcB*R22C and *phcQ*R154C mutations are not located in the known functional domains of the respective proteins (see Fig. S1A in the supplemental material). The R22C mutation does not map to the *S*-adenosyl-l-methionine-dependent methyltransferase domain of PhcB nor does the R154C mutation map to the receiver domain of PhcQ. Yet, mutations alter conserved amino acids of *Ralstonia*, *Cupriavidus*, and *Paraburkholderia* homologous proteins and would influence protein function as suggested by the Phyre2 protein structure prediction server (Fig. S1B and C) (32).

**FIG 5:**
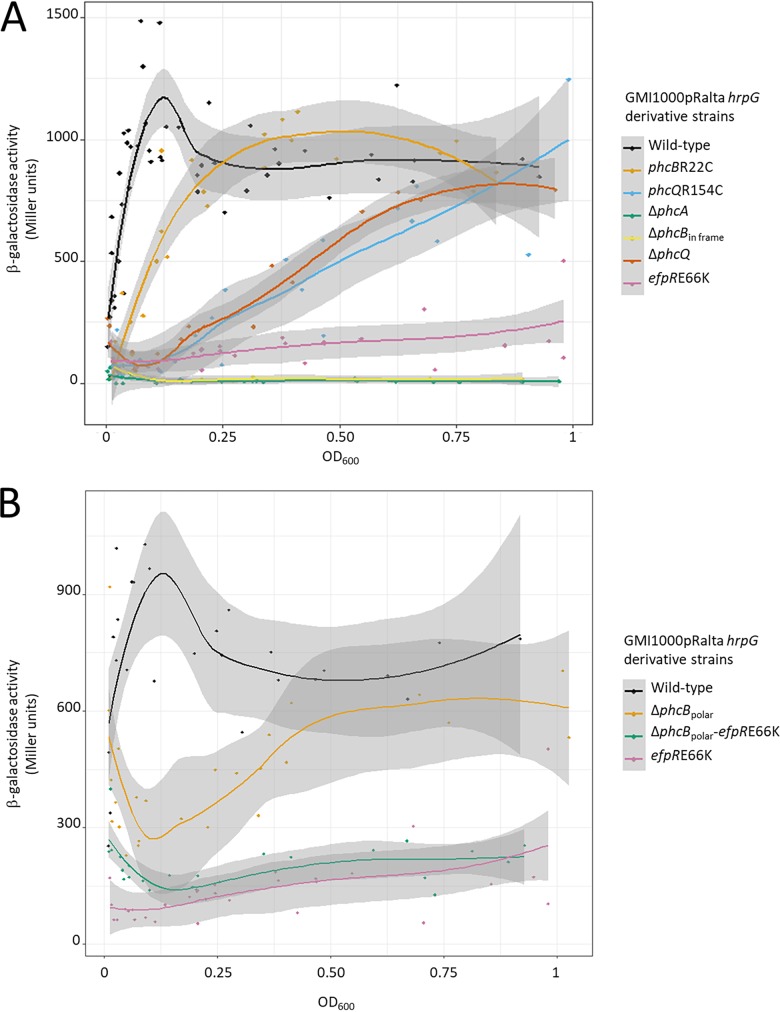
Kinetics of the p*xpsR-lacZ* expression in the chimeric R. solanacearum nodulating strain [GMI1000(pRalta) *hrpG*] and derivative mutants according to the OD_600_. β-Galactosidase activities were measured on bacteria grown in rich BG medium. Tendency curves with their 95% confidence intervals were calculated using local polynomial regressions (locally estimated scatterplot smoothing [LOESS] method). Data were from 4 to 12 independent experiments. The same set of data for *efpR*E66K was used in panels A and B.

We hypothesized that these mutations affect protein conformation, stability, or efficiency and thus may alter the dynamics of PhcA responsiveness to cell density. To test this hypothesis, we monitored the expression kinetics of the *xpsR-lacZ* reporter gene fusion in various genetic backgrounds along with growth in rich liquid medium (Fig. 5A). In the nodulating chimeric strain GMI1000(pRalta) *hrpG*, the expression of *xpsR-lacZ* sharply rose at an optical density at 600 nm (OD_600_) of 0.04 (corresponding to ca. 5 × 10^7^ CFU/ml) (see Fig. S2), reached a peak at an OD_600_ of 0.12 (ca. 10^8^ CFU/ml), and then decreased and stabilized at an OD_600_ of 0.2 (ca. 2 × 10^8^ CFU/ml). No peak of expression was observed in either the *phcB* or *phcQ* SNP mutant. In the *phcB*R22C mutant, the expression was slightly delayed and reached a maximum at an OD_600_ of 0.2 (ca. 2 × 10^8^ CFU/ml). We confirmed that the *phcB*R22C mutant does not behave like a *phcB* in-frame deletion mutant, which exhibited no PhcA activity regardless of the cell density. In contrast, the *phcQ*R154C mutant closely resembled a *phcQ* deletion mutant. In both mutants, the induction of *xpsR-lacZ* was slow and linear until an OD_600_ of 1 (ca. 10^9^ CFU/ml). These dynamics of *xpsR* expression suggested that PhcQ may be involved in a positive feedback activation loop of PhcA, implying that the *phcQ* mutation may affect the synthesis of autoinducing molecules.

10.1128/mBio.03129-19.2 FIG S2 Correspondence between the OD_600_ units and the CFU for the GMI1000(pRalta) *hrpG* strain (wild type) and its derivative mutants. Bacteria grown in rich BG medium were serially diluted and plated at different OD_600_ units per milliliter. Data are from three independent experiments. A linear regression model and the associated equation are indicated for each strain. The four strains have similar correspondences between OD_600_ units and CFU. Download FIG S2, PDF file, 0.6 MB. Copyright © 2020 Tang et al. 2020 Tang et al. This content is distributed under the terms of the Creative Commons Attribution 4.0 International license.

To substantiate whether the expression of other PhcA target genes was affected by the *phcB* and *phcQ* SNP mutations, we checked by reverse transcription-quantitative PCR (qRT-PCR) the expression of seven genes known to be positively or negatively controlled by PhcA (*egl*, *flhF*, *motA*, RSc1817, Rsp0983, RSp0178, and *xpsR*) (16–18) (see Fig. S3). For this, we harvested cells grown in rich liquid medium to an OD_600_ of around 0.1, a cell density at which the expression of *xpsR* was lower in the *phcB* and *phcQ* SNP mutants than in the wild-type strain. All these genes were differentially expressed in the *phcB*R22C, *phcQ*R154C, and Δ*phcA* mutants compared to that in the GMI1000(pRalta) *hrpG* wild-type strain. Consistently with the *xpsR* expression data, expressions of these genes were less affected in the *phcB*R22C mutant than in the *phcQ*R154C mutant.

10.1128/mBio.03129-19.3 FIG S3 Expression of PhcA target genes in the GMI1000(pRalta) *hrpG* chimeric strain and the *phcB*R22C, *phcQ*R154C, and Δ*phcA* derivative mutants. Strains were cultivated in BG medium to an OD_600_ of around 0.1. Raw expression levels were normalized by *rplM* and *rpoA* expression, and ratios of gene expression in mutants versus that in the wild-type strain were calculated. Data were obtained from three independent experiments and are presented as mean Log_2_ (ratios) ± standard deviations. Genes positively regulated by PhcA were repressed in *phcB*R22C, *phcQ*R154C, and Δ*phcA* mutants. Genes negatively regulated by PhcA were found induced in *phcB*R22C, *phcQ*R154C, and Δ*phcA* mutants. *, *P* < 0.05 versus wild-type strain (Student’s *t* test). Download FIG S3, PDF file, 0.3 MB. Copyright © 2020 Tang et al. 2020 Tang et al. This content is distributed under the terms of the Creative Commons Attribution 4.0 International license.

These results showed that the *phcB* and *phcQ* mutations delay, to different degrees, the timing of Phc quorum sensing regulation, likely by decreasing the activity of the corresponding proteins.

### The *phcB*R22C and *phcQ*R154C mutations affect the synthesis of QS molecules.

**FIG 6:**
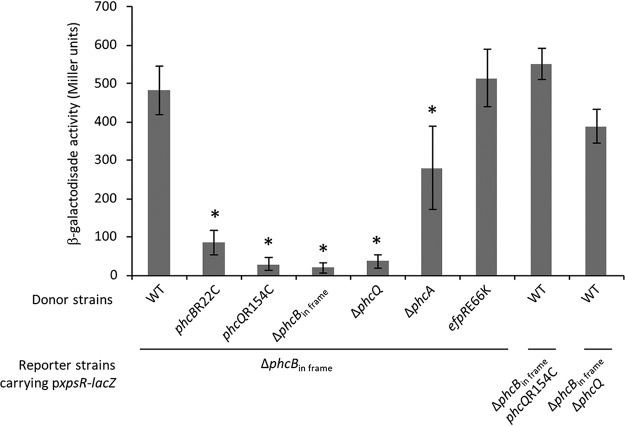
Production of QS molecules and QS signal transduction in the R. solanacearum GMI1000(pRalta) *hrpG* wild-type strain (WT) and derivative mutants. The expression of the p*xpsR-lacZ* fusion was measured in reporter strains deficient for the production of QS molecules due to a *phcB* in-frame deletion after incubation with culture supernatants from various donor strains. Supernatants were taken from liquid cultures grown to an OD_600_ of 0.1, added to 1 volume of culture of the reporter strain grown to an OD_600_ of 0.2, and incubated for 4 h before β-galactosidase activity analysis. The reporter strain Δ*phcB*_in frame_ was used to assess the ability of donor strains to produce QS molecules. Reporter strains mutated for both *phcB* and *phcQ* were used to assess the involvement of PhcQ in the transduction of QS signals. Values indicate means ± standard deviations obtained from 3 to 5 independent experiments. *, *P* < 0.01 versus the wild-type strain (Student’s *t* test).

To evaluate the efficiency of *phcB* and *phcQ* SNP mutants to produce autoinducing molecules, we set up a bioassay in which a strain deficient for the synthesis of the QS molecules [i.e., a *phcB* in-frame deletion mutant in the GMI1000(pRalta) *hrpG* background] carrying the reporter plasmidic *xpsR*-*lacZ* fusion was incubated with supernatants of cultures harvested at an OD_600_ of 0.1 from either the GMI1000(pRalta) *hrpG* wild-type strain or the *phcB*R22C, *phcQ*R154C, Δ*phcB*_in frame_, Δ*phcQ*, or Δ*phcA* derivative mutants. As expected, after 4 h of incubation with the supernatant of the wild-type strain, the *xpsR-lacZ* fusion was well expressed, while this fusion was not expressed after incubation with the supernatant of a Δ*phcB*_in frame_ mutant. In agreement with previous works showing that some PhcA targets positively control the production or the stability of 3-OH MAME (33), the *xpsR-lacZ* fusion was less expressed after incubation with the supernatant of a Δ*phcA* mutant. Interestingly, the expression of *xpsR-lacZ* was strongly reduced or near the background level after incubation with the supernatants from the *phcB*R22C, *phcQ*R154C, and Δ*phcQ* mutants (Fig. 6), indicating that the amount of active QS molecules present in the supernatants of these mutants was reduced compared to that in the supernatant of the wild-type strain taken at the same OD_600_. Moreover, we tested the involvement of PhcQ in signal transduction by incubating the double *phcQ*R154C Δ*phcB*_in frame_ or Δ*phcQ* Δ*phcB*_in frame_ mutants with the supernatant of a wild-type strain. We found that the *xpsR-lacZ* expression was not affected in these strains, indicating that PhcQ is involved in the production of QS molecules but not in the signal transduction to PhcA (Fig. 6).

### Infection-adaptive *phc* mutations alter PhcA activation during the uptake of bacteria in root hairs.

**FIG 7:**
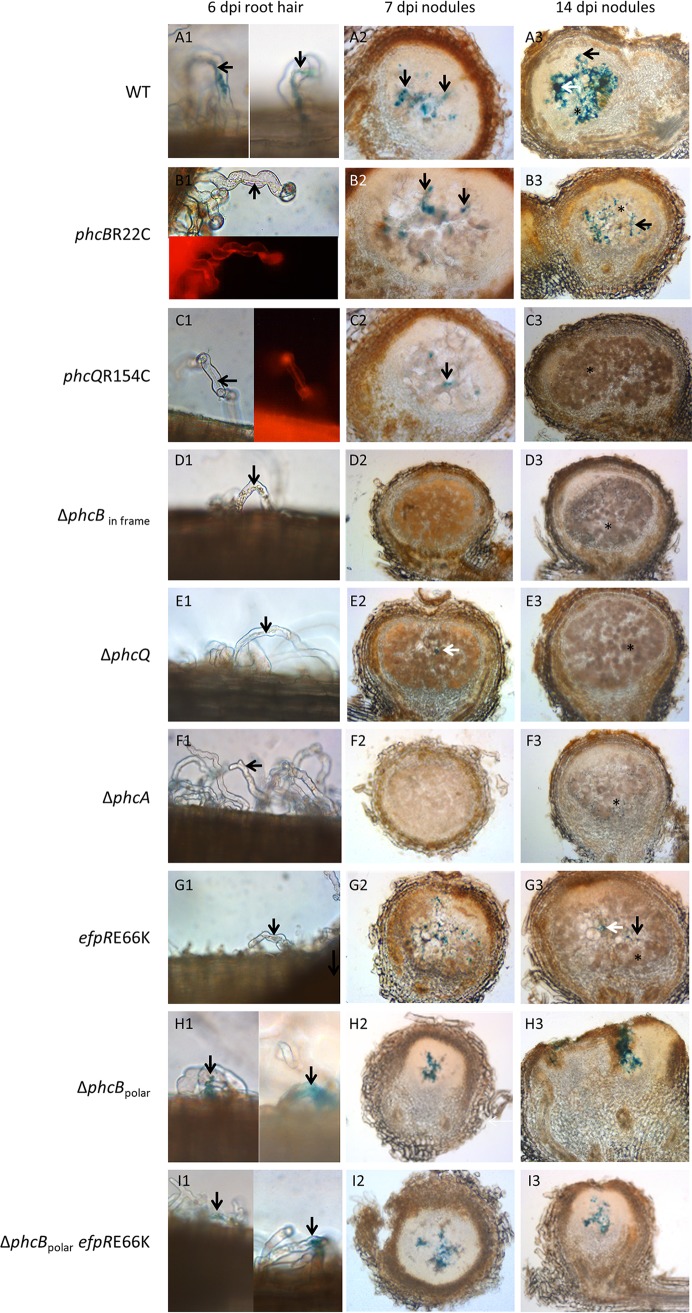
Expression of p*xpsR-lacZ* in the chimeric R. solanacearum nodulating strain [GMI1000(pRalta) *hrpG*] and derivative mutants during symbiotic interaction with *Mimosa pudica.* Roots and nodules were harvested at 6 and 7 or 14 dpi, respectively, and fixed with 2% glutaraldehyde. Roots and 55-μm sections of nodules were then stained in the presence of X-Gal (5-bromo-4-chloro-3-indolyl-β-d-galactopyranoside) to reveal beta-galactosidase activity. The *xpsR-lacZ* expression was detected in infection threads and extracellularly invaded spaces of young and mature nodules induced by all strains (A2 to C2, E2, G2 to I2, A3 to C3, G3 to I3) except by the *phcA* and *phcB*_in frame_ deletion mutants (D and F). Only the poorly infectious strains, GMI1000(pRalta) *hrpG* and its derivatives Δ*phcB*_polar_ and Δ*phcB*_polar_*-efpR*E66K mutants, expressed the fusion in root hairs (A1, H1, and I1 versus B1 to G1). The *phcB*R22C and *phcQ*R154C mutants constitutively expressed the mCherry fluorophore. Bacteria fluorescing in red can be visualized in ITs (B and C). Black arrows indicate infection threads. White arrows indicate extracellular bacteria. Light brown cells and black stars indicate intracellularly infected nodule cells. Images are representative of data obtained from at least 3 independent experiments.

In *M. pudica*, rhizobial partners enter roots via transcellular infection threads that progress toward the cortex and ultimately release bacteria within cells of the developing nodules, where they are subsequently accommodated in membrane-enclosed compartments called symbiosomes. In the evolved *Ralstonia-M. pudica* interaction, intercellular bacteria were also observed associated with necrotic zones that usually occur in nodules induced by GMI1000(pRalta) *hrpG* and occasionally in nodules induced by the *phcB* and *phcQ* SNP mutants. To know whether the delay in quorum sensing responsiveness also operates under symbiotic conditions, we assessed the expression of the *xpsR*-*lacZ* fusion at three distinct symbiotic stages, following inoculation with either GMI1000(pRalta) *hrpG* or its *phcB*R22C, *phcQ*R154C, Δ*phcB*_in frame_, Δ*phcQ*, or Δ*phcA* derivative mutants (i) in root hairs during bacterial uptake, (ii) in young nodules when bacteria are released from infection threads into the cytoplasm of plant cells, and (iii) in mature nodules accommodating intracellular bacteria, called bacteroids. As expected, in the *phcA*-deleted mutant, no expression of *xpsR-lacZ* was observed under any symbiotic conditions (Fig. 7F). Whatever the inoculated strain, no *xpsR* expression was observed in bacteroids. However, as the number of infected cells was very low in nodules induced by the GMI1000(pRalta) *hrpG* strain, it was difficult to evaluate the level of *xpsR* expression in bacteroids formed by this strain. In nodules induced by the poorly infecting strain GMI1000(pRalta) *hrpG*, *xpsR-lacZ* expression was visualized in root hair and nodule infection threads (ITs) as well as in intercellular bacteria (Fig. 7A). When roots were inoculated by any of the two *phcB* and *phcQ* SNP mutants, loss of *xpsR-lacZ* expression was only observed in ITs progressing in root hairs, *xpsR-lacZ* being normally expressed in nodule ITs (Fig. 7B and C). These results suggested that activation of PhcA in the late infection-adapted *phc* mutants was impaired during a very early symbiotic stage, corresponding to the bacterial uptake in root hairs and initiation of infection threads. This early stage thus appeared crucial for late intracellular accommodation of bacteria in nodules.

### QS modulation as an alternative adaptive path to EfpR inactivation.

The *phc* mutations have the same impact on intracellular infection as mutations in the EfpR path previously detected in other evolved lines. Indeed, the size of infected and necrotic zones in nodules induced by *phc* SNP mutants was similar to that of nodules induced by *efpR* mutants (Fig. 2) (27). To compare the adaptiveness of the *efpR* and *phc* SNP mutations in a global and more integrative way, we measured their relative *in planta* fitness following plant coinoculation. We showed that the *in planta* fitness of the *efpR*E66K mutant was not significantly different from that of the *phcB*R22C or *phcQ*R154C mutant in a GMI1000(pRalta) *hrpG* context (Fig. 4). Moreover, the double *phcB*R22C*-efpR*E66K or *phcQ*R154C*-efpR*E66K mutant was as fit as the single *efpR*E66K mutant, indicating that *efpR* and *phc* adaptive mutations do not have cumulative effects (Fig. 4). Similar to that in *phc* SNP mutants, in an *efpR*E66K mutant, the expression of *xpsR* was strongly affected but not abolished under *in vitro* conditions (Fig. 5A), while under *in planta* conditions, expression of the *xpsR-lacZ* fusion was visualized in nodule infection threads but not in root hair infection threads (Fig. 7G1).

In a previous study, we determined the EfpR regulon in a GMI1000(pRalta) *hrpG* context (27). Strikingly, this EfpR regulon largely overlaps with the PhcA regulon determined recently in three independent studies (16–18). Among the 200 putative *efpR* targets, 160 (80%) were also found differentially expressed in at least one of the PhcA regulons (see Table S2). These genes include 17 for EPS synthesis, 52 for motility, 14 for hemin/siderophore transport and metabolism genes, the *prhI* sigma factor, and 10 genes encoding Hrp and T3 effector proteins. In addition, both PhcA and EfpR were shown to be metabolic repressors (15, 17, 27, 28). The metabolic profiles of the two mutants also display high similarities, since 88% of substrates found to be more efficiently metabolized in GMI1000(pRalta) *hrpG efpR*E66K (27) were also better metabolized in a *phcA* mutant (17) (see Table S3). Hence, the EfpR path may either affect PhcA activity directly, interfere with its activating pathway, PhcBSRQ, or act on some genes commonly targeted by PhcA.

10.1128/mBio.03129-19.6 TABLE S2 Comparison of genes differentially expressed in *efpR*E66K and Δ*phcA* mutants. Download Table S2, XLSX file, 0.1 MB. Copyright © 2020 Tang et al. 2020 Tang et al. This content is distributed under the terms of the Creative Commons Attribution 4.0 International license.

10.1128/mBio.03129-19.7 TABLE S3 Carbon, nitrogen, and phosphorus sources better metabolized in the *efpR*E66K mutant. Download Table S3, XLSX file, 0.1 MB. Copyright © 2020 Tang et al. 2020 Tang et al. This content is distributed under the terms of the Creative Commons Attribution 4.0 International license.

To assess whether the EfpR pathway could interfere with the QS sensory cascade PhcBSRQ, we first determined the production of QS molecules in an *efpR*E66K mutant using the bioassay described above. We showed that the *efpR*E66K mutant produced similar amounts of active QS molecules as the wild-type GMI1000(pRalta) *hrpG* strain (Fig. 6). Then, we combined a *phcB* polar deletion mutation, which downregulated the expression of the full *phcBSRQ* operon (see Fig. S4), with an *efpR*E66K mutation in the GMI1000(pRalta) *hrpG* strain and monitored the expression of the *xpsR-lacZ* reporter gene fusion by β-galactosidase assay along growth as described above. In the single Δ*phcB* polar deletion mutant, probably because of the absence of the PhcSR repressive action (14), PhcA was constitutively active at an intermediary level throughout growth, although with some variations (Fig. 5B). Interestingly, the *xpsR-lacZ* expression in the double Δ*phcB*_polar_-*efpR*E66K mutant was always lower than in the single Δ*phcB*_polar_ mutant, indicating that the *efpR*E66K mutation can repress *xpsR* in the absence of the PhcBSRQ proteins and thus may alter the PhcA regulon in a PhcBSRQ-independent manner. Notwithstanding, when the OD_600_ was below 0.1, *xpsR-lacZ* expression was higher in the double Δ*phcB*_polar_-*efpR*E66K mutant than in the single *efpR*E66K mutant, suggesting an additive effect of the two mutations at low cell density. Consistently, both Δ*phcB*_polar_ and Δ*phcB*_polar_-*efpR*E66K mutants were poorly infectious (Fig. 2) and harbored a detectable *xpsR-lacZ* expression in epidermal ITs (Fig. 7H1 and I1), thus reinforcing the correlation between the absence of early *xpsR* expression in root hair ITs and a nice late intracellular infection phenotype.

10.1128/mBio.03129-19.4 FIG S4 Expression of *phcS*, *phcR*, and *phcQ* genes in the GMI1000(pRalta) *hrpG* chimeric strain and its Δ*phcB* polar derivative mutant. Strains were cultivated in BG medium until mid-exponential phase. Raw expression levels were normalized by *rplM* expression, and ratios of gene expression in mutants versus that in the wild-type strain were calculated. Data were obtained from three independent experiments and are presented as Log_2_ (mean ratios) ± standard deviations. *, *P* < 0.01 versus the wild-type strain (Student’s *t* test). Download FIG S4, PDF file, 0.6 MB. Copyright © 2020 Tang et al. 2020 Tang et al. This content is distributed under the terms of the Creative Commons Attribution 4.0 International license.

### *xpsR* deletion did not improve intracellular infection.

To assess whether the downregulation of *xpsR* itself could solely account for the infection phenotype of the *efpR* and *phc* mutants, we constructed a *xpsR* deletion mutant in the GMI1000(pRalta) *hrpG* background and quantified its nodule cell infection ability. This mutation did not improve the intracellular infection (Fig. 2), suggesting that either EPS biosynthesis is not involved in infection or repression of EPS biosynthesis alone is not sufficient to improve infection.

### Quorum sensing modulation decreased the pathogenicity of R. solanacearum.

**FIG 8:**
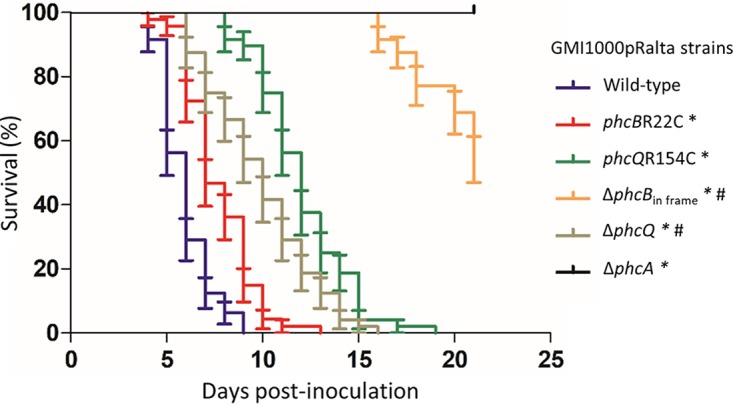
Disease symptoms induced by wild-type and mutant strains of the chimeric *Ralstonia* on Arabidopsis thaliana. Kaplan-Meyer survival analysis of *A. thaliana* plants inoculated by the wild-type GMI1000(pRalta) chimeric *Ralstonia* and its *phcB*R22C, *phcQ*R154C, Δ*phcB*_in frame_, Δ*phcQ*, and Δ*phcA* derivative mutants. *, the survival curves are significantly different from the wild-type curve; #, the survival curves are significantly different from the corresponding *phc* mutant curves (*P* < 0.001, Gehan-Breslow-Wilcoxon test). Values were obtained from 3 independent series of 16 plants.

To evaluate whether the *phc* mutations adaptive for *M. pudica* nodule cell infection affect the pathogenicity of R. solanacearum, we introduced the *phcB*R22C, *phcQ*R154C, Δ*phcB*_in frame_, Δ*phcQ*, and Δ*phcA* mutations into the pathogenic GMI1000(pRalta) strain and followed the disease symptoms over 20 days on the host plant Arabidopsis thaliana. While the *phcA* deletion mutant induced no symptom on *A. thaliana*, both *phc* SNP mutants induced significantly delayed symptoms, *phcB*R22C being delayed by ca. 1 to 2 days and *phcQ*R154C being delayed by ca. 6 to 7 days compared with that in the wild-type strain (Fig. 8). The Δ*phcQ* mutant appeared slightly more virulent than the *phcQ*R154C mutant. In contrast, the Δ*phcB*_in frame_ mutant induced symptoms strongly delayed, by ca. 14 days, compared with that in the *phcB*R22C mutant strain (Fig. 8), suggesting that PhcA may have residual activity in the absence of QS molecules.

## DISCUSSION

Quorum sensing is a widespread bacterial regulatory system allowing rapid shifts in behavior in response to changes in cell density or confinement (34). The mode and tempo of signal transduction and target outputs of each quorum sensing system reflect the unique biology carried out by a particular bacterial species. Here, we show that a system optimized to allow survival in a specialized niche can be rapidly redesigned to promote thriving in a very different environment, i.e., change in QS responsiveness helps the extracellular plant pathogen R. solanacearum having acquired a set of essential symbiotic genes to turn into an intracellular legume symbiont. During experimental evolution under legume (*M. pudica*) selection pressure, point mutations affecting two components of the Phc QS regulatory cascade, the autoinducer synthase PhcB and the regulatory protein PhcQ, modified the kinetics of activation of the downstream central regulator PhcA. The delay in PhcA activation in the *Ralstonia phc*-mutated evolved clones observed *in vitro* results in an inactivation of PhcA in epidermal ITs, which correlates with their nice nodule intracellular infection phenotypes on *M. pudica*. Conversely, these mutations, when introduced in the *Ralstonia* ancestor, reduced the virulence capacity of the strain, reemphasizing the antagonism between pathogeny and symbiosis (24, 26, 27). Unlike other infection-adaptive mutations we previously identified (*hrpG* and *efpR*) (24, 26, 27), the virulence regulator PhcA was not constitutively inactivated. Potentially, this conditional inactivation could be more favorable for subsequent adaptation steps. Interestingly, in Cupriavidus taiwanensis, a *Ralstonia*-neighboring taxon that evolved into *Mimosa* symbionts, the *phcB* and *phcS* orthologous genes were found under positive selection and associated with the transition to symbiosis during the natural evolution of this rhizobium species (29), likely reflecting the need to adjust QS to the legume endosymbiotic niche. Adaptive mutations altering QS responsiveness were also evidenced during experimental and natural evolution of other pathogenic and symbiotic bacteria such as Vibrio fischeri and Staphylococcus aureus (35–38), exemplifying the role of these sensory systems in bacterial adaptation and phenotypic diversification. The role of QS in rhizobial symbiosis is, however, variable, either neutral (39), positive (40–42), or negative (43, 44). Rhizobial QS systems antagonistically control EPS biosynthesis and motility-chemotaxis but also affect a wide spectrum of other physiological traits that vary among rhizobial species, including biofilm formation, swarming motility, the type III secretion system, plasmid transfer, cell division, metabolism, and transport (6, 39, 45).

**FIG 9:**
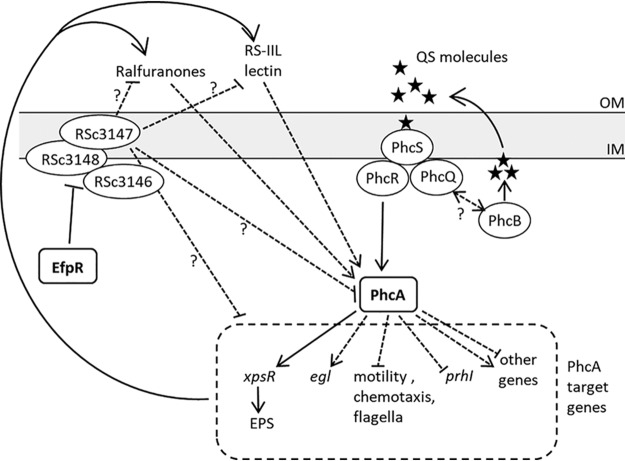
Possible connections between PhcA and EfpR regulatory pathways. PhcA is activated by the PhcBSRQ quorum sensing sensory cascade. Above the QS threshold of ca. 5 × 10^7^ cell/ml, PhcA is rapidly activated. The regulatory protein PhcQ contributes to the dynamics of activation of PhcA by influencing the synthesis of the QS molecules. EfpR represses the Rsc3146 to -3148 genes. In an *efpR* mutant, the strong induction of Rsc3146 to -3148 genes affects a large part of the PhcA regulon either by modulating PhcA activity, by acting on the expression of some PhcA target genes, or by inhibiting one of the positive feedback loops of activation of PhcA (16, 33). Dashed lines indicate potential indirect effects.

Studies identifying the molecular mechanisms that underlie convergent evolution have shown that mutations involved in a given phenotype often target the same pathways, making experimental evolution a powerful tool to decipher regulatory networks (27, 46). In this line, our experiment allowed us to uncover a new component of the R. solanacearum Phc QS system and provided additional information on the functioning of the Phc network. Although the PhcBSR(Q) pathway was identified approximately 20 years ago (14), the roles of several components were not clearly elucidated. This was the case for the regulatory protein PhcQ, which was suspected to be involved in the QS cascade because it belongs to the PhcBSRQ operon. Here, we demonstrated that PhcQ is involved in the dynamics of activation of PhcA in response to the quorum. In addition, the synthesis of QS molecules was found to be affected in a *phcQ* mutant, suggesting that the activity of the autoinducer synthase PhcB is directly or indirectly regulated by PhcQ. The PhcQ protein is composed of a receiver domain and an uncharacterized C-terminal part. Several different mechanisms of action have been reported for proteins containing a single-receiver domain, including a direct regulatory effect on the activity of a downstream protein, the transfer of phosphate in phosphorelays, or a role as phosphate-sinks that redirect phosphate flux away from histidine kinases (47). It is also possible that PhcQ, through its receiver domain, interacts with PhcB and modulates its activity. Such a role in the synthesis and transduction of a diffusible signal factor (DSF) has been demonstrated for the receiver domain of the Xanthomonas campestris sensor kinase RpfC in a QS cascade (48). In a previous study, we showed that in some evolved *Ralstonia* lines, massive nodule cell infection capacity was gained through the inactivation of another central regulator, EfpR, controlling the membrane associated RSc3146 to -3148 genes (27). The *phc* and *efpR* mutations displayed similar *in planta* fitness, and their combination had no cumulative effect on this fitness. This, together with strong similarities in the transcriptomic and metabolomic profiles of the two regulators, suggested a connection between the EfpR and PhcA regulatory pathways. Our findings indicated that the EfpR regulatory cascade does not interfere with the PhcBSRQ-mediated QS signal transduction and thus that the two paths act independently on the PhcA regulon. However, the mechanism by which the EfpR-RSc3146-RSc3148 pathway represses the PhcA regulon remains unknown. It may either repress PhcA activity, act independently on PhcA target genes, or impair one of the positive feedback loops activating PhcA (16, 33) (Fig. 9).

In rhizobium-legume symbiosis, the accommodation of bacteria within nodule cells is a crucial step, since it protects internalized bacteria from plant defense reactions, while massive bacterial multiplication allows for nitrogen fixation levels that sustain plant growth (49). How this property is acquired during evolution is only partly understood. An intrinsic infection capacity is gained through the acquisition of *nod* genes that determine the synthesis of Nod factors. These lipochitooligosaccharide signal molecules specifically recognized by plant LysM receptors trigger the developmental program required for nodule organogenesis, bacterial infection (50, 51), and partial suppression of plant defenses (52). Other bacterial factors, such as surface polysaccharides, are likely recruited in recipient genomes to achieve infection. In Lotus japonicus, for instance, compatible bacterial EPS are specifically recognized by the plant LysM receptor kinase EPR3, which might activate a signaling cascade that controls and maintains an intracellular infection mode (53–55). In this study, we showed that inactivation of the central regulator PhcA was an alternative way to the inactivation of another global regulator, EfpR, to improve intracellular infection during experimental evolution of R. solanacearum into legume symbionts. Since PhcA and EfpR control a common set of more than 150 genes, including those for exopolysaccharides and motility and virulence factors, it is likely that the concerted change of expression of several genes rather than a single gene is involved in infection improvement. Indeed, the sole inactivation of XpsR, the activator of EPS biosynthesis genes, did not lead to an improved infection phenotype. Other virulence genes controlled by these systems may trigger plant defense reactions and thus hamper symbiosis establishment (16–18). Another common interesting feature of both regulators is that their repression/inactivation enhances the metabolism of the bacterium, increasing its growth rate on many substrates. Future investigation of other intracellular lines, where EfpR and Phc pathways do not seem targeted by evolution, may narrow the number of possible functions responsible for phenotypic changes. Yet, we were able to identify at which symbiotic stage modifications were required, by monitoring the *in planta* expression of a direct PhcA target, *xpsR*. A clear change induced by the *phc* mutations was a specific loss of *xpsR* expression in infection threads of root hairs, suggesting that this step may condition the success of the subsequent accommodation of bacteria within nodule cells. The link between early and late events of the infection process is supported by the fact that several plant genes (NFP/NFR5, LYK3/NFR1, SYMRK, or IPD3/CYCLOPS) involved in early symbiotic signaling in the root epidermis are also required for the release of bacteria in nodule cells (56–58). Furthermore, a tight coordination between the progression of ITs and the periclinal divisions of specific root cell layers was proposed to determine the intracellular infection of nodules (59). It is thus possible that, for either signaling or metabolic reasons, the primary ITs formed by *efpR* and *phc* mutants progress more rapidly than those made by a single *hrpG* mutant, allowing a better invasion of nodule cells. Future fine cellular analyses of the progression rate of ITs as well as a comparison of molecular plant responses differently induced by poor and nice infectious evolved clones should help us to understand intracellular infection, the distinctive feature of nitrogen-fixing root nodule symbioses and possibly the first trait acquired by the common ancestor of the root nodule forming plant clade FaFaCuRo (Fabales, Fagales, Cucurbitales and Rosales) during evolution (60).

## MATERIALS AND METHODS

### Bacterial strains, plasmids, and growth conditions.

Bacterial strains and plasmids used in this work are listed in Table S1 in the supplemental material. R. solanacearum strains were grown at 28°C either on rich BG medium (61) or on MP minimal medium (62) supplemented either with 20 mM glutamate for gene expression analyses or with glycerol 2% for bacterial transformation. Antibiotics were used at the following concentrations: trimethoprim, 100 μg/ml; spectinomycin, 40 μg/ml; kanamycin, 50 μg/ml; tetracycline, 10 μg/ml.

10.1128/mBio.03129-19.5 TABLE S1 Strains and plasmids used in this study. Download Table S1, DOCX file, 0.1 MB. Copyright © 2020 Tang et al. 2020 Tang et al. This content is distributed under the terms of the Creative Commons Attribution 4.0 International license.

### Plants assays and cytological analyses.

*Mimosa pudica* seedlings from Australian origin (B&T World Seed, Paguignan, France) were cultivated as previously described (29).

For cytology, nodules were harvested at 21 days postinoculation (dpi) and cut into 55-μm sections with a vibrating blade microtome (VT1000 S; Leica, Wetzlar, Germany). For each nodule, longitudinal sections were observed using an inverted microscope (DM IRB/E; Leica, Wetzlar, Germany), and images were acquired using a charge-coupled-device (CCD) camera (Color Coolview; Photonic Science, Milham, UK). Quantification of infection and necrosis areas on nodule sections was performed as described previously (25, 30) using the Image-Pro Plus software and based on the HIS method (Media Cybernetics, Rockville, MD, USA). Necrotic areas are characterized by a dark-brown color, while intracellularly infected cells are characterized by a light-brown color. Results of infection and necrosis quantifications were obtained from 2 to 3 independent experiments for each strain. At least 16 plants were analyzed per experiment.

For relative *in planta* fitness assays, we coinoculated *M. pudica* plants with a pair of different strains in equivalent proportions (5 × 10^5^ bacteria of each strain per plant). Coinoculated strains were carrying the same antibiotic marker and expressing or not the mCherry fluorophore. Nodules from 20 plants were harvested 21 days after inoculation, pooled, surface sterilized, and crushed together. Dilutions of nodule crushes were spread on solid medium. In cases where one of the coinoculated strains was expressing the mCherry fluorophore, colonies formed by each strain were counted under a stereo zoom microscope under bright-field and fluorescence illuminations (AxioZoom V16; Zeiss, Oberkochen, Germany). In cases where both coinoculated strains were not expressing any fluorophores (comparison of Δ*phcA* mutant with *phcB*R22C or *phcQ*R154C mutant), 96 colonies from the nodule crushes were screened by PCR for the presence/absence of mutations. Three independent experiments were performed for each comparison.

### Beta-galactosidase assays.

Expression of the plasmidic *xpsR-lacZ* fusion was assessed by β-galactosidase assay of 100-μl aliquots of bacterial cultures grown in rich BG medium. Samples were collected either at an OD_600_ of 0.2 (Fig. 3) or every 2 to 3 h along a growth curve from an OD_600_ of 0.005 to an OD_600_ of 1. β-Galactosidase activities were measured in Miller units as described previously (63). The correspondence between the OD_600_ units and the number of bacteria (CFU) was determined for each strain throughout growth in rich BG medium by plating serial dilutions of cultures (Fig. S2).

To test the production of QS molecules by the chimeric strains, the GMI1000(pRalta) *hrpG* Δ*phcB*_in frame_ strain unable to produce QS molecules and expressing the reporter gene fusion p*xpsR*-*lacZ* was grown in rich BG medium until the OD_600_ reached ca. 0.2. In parallel, strains producing QS molecules [GMI1000(pRalta) *hrpG* and its *phcB*R22C, *phcQ*R154C, Δ*phcB*_in frame_, Δ*phcQ*, Δ*phcA*, and *efpR*E66K derivative mutants) were grown in rich BG medium until the OD_600_ reached 0.1. Supernatants from cultures of QS molecule donor strains were prepared by centrifugation for 15 min at 5,000 rpm followed by filtration at 0.22 μm. One volume of filtered supernatant was added to 1 volume of culture of the reporter strain and incubated for 4 h at 28°C under agitation before β-galactosidase activity analysis. β-Galactosidase activities were measured from 100-μl aliquots of bacterial cultures. Background expression level of the *xpsR*-*lacZ* reporter fusion was measured by incubating the reporter strain with the supernatant of a culture of the GMI1000(pRalta) *hrpG* Δ*phcB*_in frame_ strain.

To test the QS signal transduction in the *phcQ*R154C and Δ*phcQ* mutants, a similar experiment was performed using either the GMI1000(pRalta) *hrpG phcQ*R154C Δ*phcB*_in frame_ p*xpsR*-*lacZ* or GMI1000(pRalta) *hrpG* Δ*phcQ* Δ*phcB*_in frame_ p*xpsR*-*lacZ* strains as reporter strains and the GMI1000(pRalta) *hrpG* as donor strain.

### Statistical analyses.

Statistical analyses of infection and necrosis sizes of nodule sections were performed using R software (version 3.5.1) and the nonparametric multiple-comparison test of Kruskal-Wallis. For the comparisons of *in planta* relative fitness, β-galactosidase activities in mid-exponential-phase cultures, and gene expression, we did two-sided *t* tests. The local polynomial regressions and confidence intervals of β-galactosidase activities throughout growth were performed using the R package ggplot2 and the geom_smooth function (64).

Other materials and methods concerning genome resequencing and detection of mutations in evolved clones, constructions of mutants and plasmids, detection of *xpsR-lacZ* expression *in planta*, gene expression analyses by quantitative reverse transcription-PCRs, pathogenicity assays, protein structure prediction, and protein alignments are provided in Text S1 in the supplemental material.

10.1128/mBio.03129-19.8 TABLE S4 Primers used in this study. Download Table S4, XLSX file, 0.1 MB. Copyright © 2020 Tang et al. 2020 Tang et al. This content is distributed under the terms of the Creative Commons Attribution 4.0 International license.
