# Supplementary material for: Modulation of Quorum Sensing as an Adaptation to Nodule Cell Infection during Experimental Evolution of Legume Symbionts
Source: mBio. 2020 Jan 28;11(1):e03129-19. doi: 10.1128/mBio.03129-19 (PMC6989110; doi:10.1128/mBio.03129-19)
Supplement: FIG S1 [file mBio.03129-19-sf001.pdf]

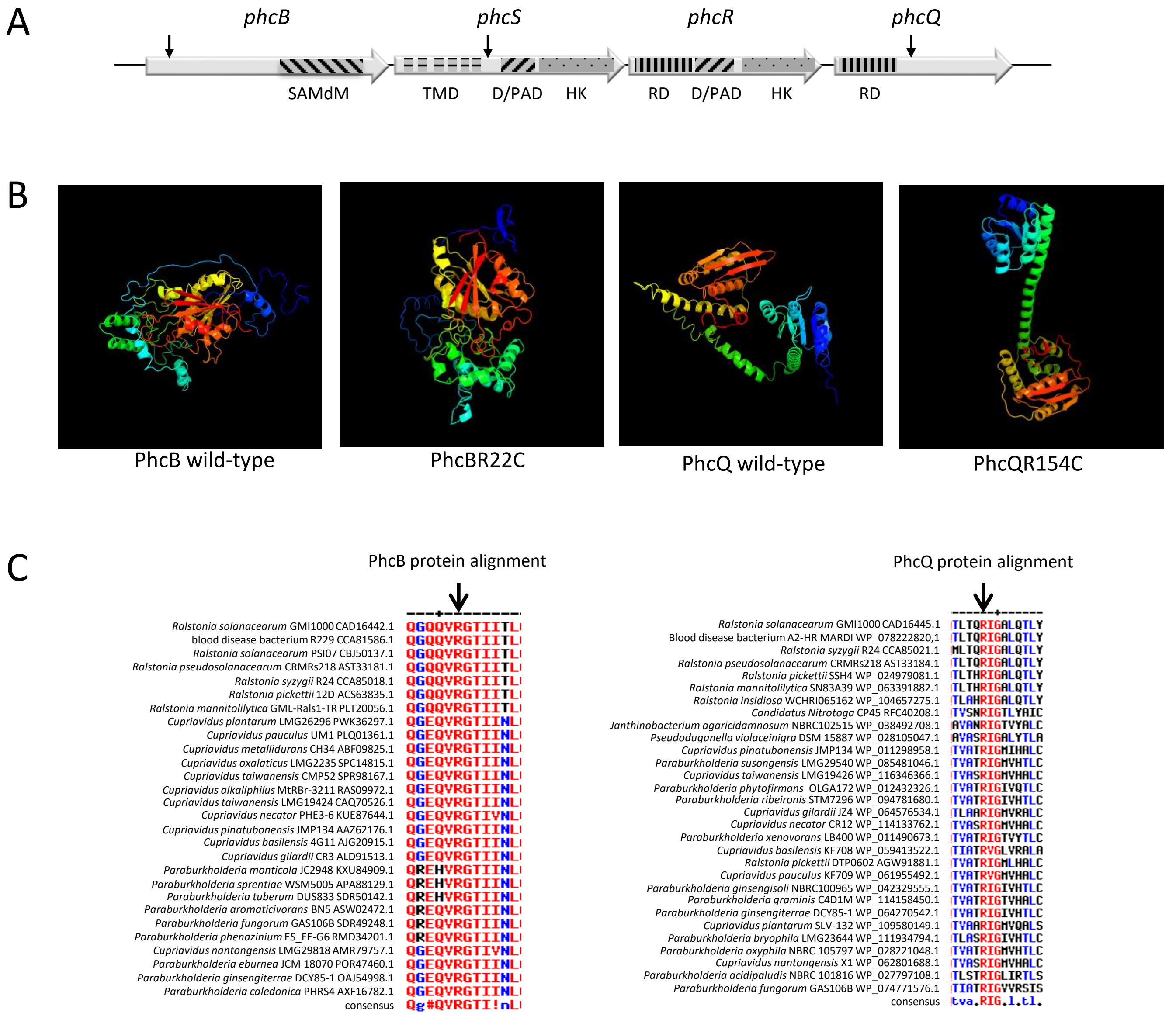

Figure S1. A. Representation of the *R. solanacearum phcBSRQ* operon and structural domains of the corresponding proteins. SAMdM, S-adenosyl-L-methionine-dependent methyltransferase. TMD, transmembrane domains. D/PAD, dimerisation/phosphoacceptor domain. HK, histidine kinase. RD, receiver domain. Arrows indicate the mutated positions in *phcB* (R22C), *phcS* (L161R) and *phcQ* (R154C).

B. Prediction of the 3-dimensional structures of the PhcB and PhcQ wild-type and mutant proteins. Structures were predicted using the Phyre [2] server (Kelley LA, Mezulis S, Yates CM, Wass MN, Sternberg MJ, Nat Protoc 10:845-58, 2015).

C. Protein alignments showing the conservation of the R22 and R154 amino acids of the PhcB and PhcQ proteins respectively. The amino acid sequences of PhcB/Q from *R. solanacearum* GMI1000 were blasted against the non-redundant NCBI database to obtain a list of homologs from other organisms with identity ranging from 64% to 100% for PhcB and 53% to 100% for PhcQ. A subset of hits, covering the diversity of genus and species containing *phcB* and *phcQ* homologs, is presented.

Sequences were aligned using MultAlin (http://multalin.toulouse.inra.fr/multalin/multalin.html).

Color red indicates amino acid residues highly conserved (>90%), color black means positions neutrally conserved (50%-90%), while color blue indicates positions lowly conserved (<50%).
