# Supplementary material for: Modulation of Quorum Sensing as an Adaptation to Nodule Cell Infection during Experimental Evolution of Legume Symbionts
Source: mBio. 2020 Jan 28;11(1):e03129-19. doi: 10.1128/mBio.03129-19 (PMC6989110; doi:10.1128/mBio.03129-19)
Supplement: TEXT S1 [file mBio.03129-19-s0001.docx]

**Supplementary materials and methods**

**Genome resequencing and detection of mutations in evolved clones**

Evolved clones were re-sequenced either by C.E.A/IG/Genoscope using the Illumina GA2X technology (clones E8, K8 and M8), or by the GeT platform (https://get.genotoul.fr/) using the Illumina technology HiSeq2000 (clones M4, M12), or MiSeq (clones E12, E13 and K7), or HiSeq3000 (clones E4, E7, K1 to K6, K9 to K15, M2, M6, M10, M14). High throughput sequencing data were analyzed as described previously (1) with the PALOMA bioinformatics pipeline implemented in the Microscope platform (2). The complete list of mutational events generated for all the clones from this study are available on the Microscope platform (<https://www.genoscope.cns.fr/agc/microscope/expdata/>NGSProjectEvo.php, SYMPA tag).

The presence of the *phcB*R22C, *phcQ*R154C, and *phcS*L161R mutations was verified in all evolved clones of the E, K, and M bacterial lineages either by PCR amplification and Sanger sequencing of PCR products or by direct PCR screening using couples of primers specifically amplifying wild-type or mutated alleles (see construction of bacterial strains below and Table S4 for primer sequences).

**Construction of bacterial strains and plasmids**

The sequences of oligonucleotides used in bacterial strain construction are listed in Table S4.

The punctual mutations, *phcB*R22C, *phcQ*R154C, and *phcS*L161R, and corresponding wild-type alleles were introduced in *R. solanacearum* genomes using the MuGENT technique firstly described by (3) and used by (4). Briefly, one fragment carrying an antibiotic resistance marker was co-transformed with a 6-Kb PCR product carrying the point mutation or the wild-type allele to introduce. Antibiotic resistance DNA markers used were the *Sca*I-linearized plasmids, either pRCK-P*ps*-GFP, or pRCK-P*ps*-mCherry (4), or pCBM142 (1), which allows the chromosomal integration of the antibiotics (kanamycin or spectinomycin) resistance gene and the constitutive P*psbA*-GFP/mCherry fusions in the intergenic region downstream *glmS*. The 6 kb regions surrounding the mutation position were obtained by PCR amplification using genomic DNA of evolved clones as template and high fidelity Phusion DNA polymerase (ThermoFisher Scientific, Waltham, Massachusetts, USA). Competent cells of *R. solanacearum* were transformed with 3 µg of PCR products and 300 ng of marked DNA. Transformants were selected on antibiotics and screened by PCR for the presence of the mutation using two couples of primers specific to wild-type and mutated alleles respectively. Screening primers were designed with a mismatched nucleotide at the 3’ penultimate position and either the wild-type or the mutated nucleotide at the 3’ end.

To delete *phcA,* *phcB* (polar and non-polar deletions), and *phcQ*, upstream and downstream fragments of each gene were amplified by PCR using the high fidelity Phusion DNA polymerase (ThermoFisher Scientific). PCR fragments were A-tailed using 0.2 mM dATP and 5 units of GoTaq DNA polymerase (Promega, Madison, Wisconsin, USA) prior ligation into the pGEM-T vector (Promega). To inactivate PhcA or delete PhcB polarly, the downstream fragments were digested from the pGEM-T plasmids by *Hin*dIII and *Bam*HI and cloned into the pGEM-T plasmids carrying the upstream fragment digested with the same restriction enzymes. Then a *Hin*dIII-digested spectinomycin resistance cassette obtained from pHP45Ω (5) was inserted in the *Hin*dIII restriction site located between the upstream and downstream fragments. Resulting plasmids were linearized by *Sca*I and introduced into the chimeric *Ralstonia* strains by natural transformation. Transformants were selected on spectinomycin and verified by PCR. To delete *phcQ and phcB* in frame, fragments were digested from pGEM-T plasmids by *Xho*I and *Bam*HI for the *phcQ* upstream fragment, by *Bam*HI and *Xba*I for the *phcQ* downstream fragments, by *Xho*I and *Eco*RI for the *phcB* upstream fragment, and by *Eco*RI and *Xba*I for the *phcB* downstream fragment. Digested upstream and downstream fragments for each gene were then cloned together into the pEX18Tc, a suicide plasmid carrying the *sacB* selection gene (6), digested by *Xho*I and *Xba*I. Resulting plasmids were introduced into the chimeric *Ralstonia* strains by triparental conjugation using the pRK600 as helper plasmid. Transconjugants were first selected on tetracycline, verified for the first event of integration then grown on non-selective liquid BG medium, and plated on BG medium supplemented with 5% sucrose to excise the suicide plasmid by a second event of recombination. Tetracyclin-sensitive deletion mutants were screened and deletion was checked by PCR. The polar effect of the *phcB* deletion was verified by qRT-PCR on bacteria grown to mid-exponential phase in rich BG medium (Fig. S4).

To construct the p*xpsR-lacZ* plasmidic fusion, a 810 bp fragment upstream the start codon of *xpsR* was amplified by PCR with the oCBM2880-oCBM2881 primer pair. The fragment was digested with *Hin*dIII and *Pst*I restriction enzymes and cloned into the pCZ388 (7) into the same restriction sites. The resulting pCBM181 (p*xpsR*-*lacZ*) and the empty pCZ388 plasmids were introduced into *R. solanacearum* chimeric strains by conjugation using the pRK600 as helper plasmid.

To inactivate the *xpsR* gene, a DNA fragment surrounding the gene was amplified by PCR using the oCBM982-oCBM983 primer pair. The fragment was digested by the *Eco*RI and *Spe*I restriction enzymes and cloned into the pBBR1MCS-5 vector digested by the same enzymes. The spectinomycin resistance cassette was prepared from the pHP45Ω plasmid and inserted within the *Bam*HI restriction site located in the *xpsR* gene. The resulting plasmid was linearized by *Eco*RI and transformed into *Ralstonia* chimeric strains. The transformants were verified by PCR using the oCBM1006-oCBM1007 primer pair.

**Detection of *xpsR-lacZ* expression *in planta***

To detect the *xpsR-lacZ* expression in early symbiotic stages, roots were collected at 6 dpi, fixed with 2% glutaraldehyde for 1.5 h under vacuum, then washed several times with Z′ buffer (0.1 M potassium phosphate buffer (pH 7.4), 1 mM MgSO4, and 10 mM KCl). Fixed roots were stained for 1 h at room temperature under vacuum in Z′ buffer containing 0.08% 5-bromo-4-chloro-3-indolyl-β-d-galactoside (X-gal), 5 mM K_3_Fe(CN)_6_, and 5 mM K_4_Fe(CN)_6_ then incubated for 1 more hour at 37 °C. To detect *xpsR-lacZ* expression in nodules, nodules were harvested at 7 or 14 dpi, fixed with 2% glutaraldehyde for 1.5 h under vacuum, and washed in Z’ buffer. Fixed nodules were included in 4% agarose gel, and then cut into 55 µm sections with a vibrating blade microtome (VT1000 S, Leica) before staining for 1 hour at 37 °C. Roots and nodule sections were observed under an inverted microscope (DM IRB/E, Leica) and images acquired using a CCD camera (Color Coolview, Photonic Science).

**Gene expression analyses by quantitative Reverse Transcription-Polymerase Chain Reactions (qRT-PCR)**

Bacterial cultures were arrested by adding 1/10 volume of cold phenol/ethanol (5/95) mixture, harvested by centrifugation for 10 min at 4 °C at 4,000 rpm and bacterial pellets were frozen in liquid nitrogen and stored at -80 °C until RNA extraction. Pellets were resuspended in 1 mg/ml of lysozyme in Tris/EDTA (10/1, pH 8) buffer and incubated for 5 min at room temperature prior RNA extraction. RNAs were extracted using the mirVana kit (ThermoFisher Scientific), treated with Turbo DNase for 30 min at 37 °C (ThermoFisher Scientific) to remove residual genomic DNA, and then purified with the Sureprep kit (ThermoFisher Scientific). RNA concentration was determined using a Nanodrop ND1000 spectrophotometer (ThermoFisher Scientific). RNA quality was assessed using a 2100 Bioanalyzer instrument (Agilent Technologies, Santa Clara, California, USA). 1 μg of RNA were reverse transcribed using the Transcriptor Reverse Transcriptase kit (Roche, Life technologies, Carlsbad, California, USA) and random hexamers as primers. Real-time PCRs were run on a LightCycler system (Roche, Life technologies) using the LightCycler 480 SYBR Green I Master kit (Roche, Life technologies) according to manufacturer’s instructions. Primer sequences used for qRT-PCR are listed in Table S4.

**Pathogenicity assays**

*Arabidopsis thaliana* seeds from the susceptible ecotype Col-0 were sterilized, germinated, grown in Jiffy peat pellets for 3 weeks, and inoculated after root clipping as previously described (8). Disease symptoms were followed for 20 days and scored according to the disease index scale used in (4): healthy plants (score 0), 1–25% leaves wilted (score 1), 26–50% leaves wilted (score 2), 51–75% leaves wilted (score 3), and >75% leaves wilted (score 4). Each virulence assay was repeated 3 times independently with 16 plants per strain and per assay. Kaplan–Meier survival curves were plotted by transforming the symptom scores into a binary index: 0 for<75% of wilted leaves and 1 for ≥75% wilted leaves. Survival curves were compared by the Gehan–Breslow–Wilcoxon method as described (9) using GraphPad Prism version 5.03 for Windows (GraphPad software, La Jolla, California, USA).

**Protein structure prediction and protein alignments**

The 3 dimensional structures of the wild-type and mutated proteins PhcB and PhcQ were predicted using the Phyre^2^ server in intensive modeling mode (10). Conservation of the R22 and R154 amino acids of PhcB and PhcQ respectively were searched by BlastP against the non-redundant protein database from NCBI. Multiple alignments of homolog proteins were performed using the MultAlin software (11).

**Supplementary references**

1. Remigi P, Capela D, Clerissi C, Tasse L, Torchet R, Bouchez O, Batut J, Cruveiller S, Rocha EP, Masson-Boivin C. 2014. Transient hypermutagenesis accelerates the evolution of legume endosymbionts following horizontal gene transfer. PLoS Biol 12:e1001942.

2. Vallenet D, Belda E, Calteau A, Cruveiller S, Engelen S, Lajus A, Le Fevre F, Longin C, Mornico D, Roche D, Rouy Z, Salvignol G, Scarpelli C, Smith AAT, Weiman M, Medigue C. 2013. MicroScope-an integrated microbial resource for the curation and comparative analysis of genomic and metabolic data. Nucleic Acids Res 41:E636-E647.

3. Dalia AB, McDonough E, Camilli A. 2014. Multiplex genome editing by natural transformation. Proc Natl Acad f Sci U S A 111:8937-8942.

4. Capela D, Marchetti M, Clérissi C, Perrier A, Guetta D, Gris C, Valls M, Jauneau A, Cruveiller S, Rocha EPC, Masson-Boivin C. 2017. Recruitment of a lineage-specific virulence regulatory pathway promotes intracellular infection by a plant pathogen experimentally evolved into a legume symbiont. Mol Biol Evol 34:2503-2521.

5. Prentki P, Krisch HM. 1984. *In vitro* insertional mutagenesis with a selectable DNA fragment. Gene 29:303-313.

6. Hoang TT, Karkhoff-Schweizer RR, Kutchma AJ, Schweizer HP. 1998. A broad-host-range Flp-FRT recombination system for site-specific excision of chromosomally-located DNA sequences: application for isolation of unmarked *Pseudomonas aeruginosa* mutants. Gene 212:77-86.

7. Cunnac S, Boucher C, Genin S. 2004. Characterization of the cis-acting regulatory element controlling HrpB-mediated activation of the type III secretion system and effector genes in *Ralstonia solanacearum*. J Bacteriol 186:2309-2318.

8. Deslandes L, Pileur F, Liaubet L, Camut S, Can C, Williams K, Holub E, Beynon J, Arlat M, Marco Y. 1998. Genetic characterization of RRS1, a recessive locus in *Arabidopsis thaliana* that confers resistance to the bacterial soilborne pathogen *Ralstonia solanacearum*. Mol Plant Microbe Interact 11:659-67.

9. Remigi P, Anisimova M, Guidot A, Genin S, Peeters N. 2011. Functional diversification of the GALA type III effector family contributes to *Ralstonia solanacearum* adaptation on different plant hosts. New Phytol 192:976-987.

10. Kelley LA, Mezulis S, Yates CM, Wass MN, Sternberg MJ. 2015. The Phyre2 web portal for protein modeling, prediction and analysis. Nat Protoc 10:845-58.

11. Corpet F. 1988. Multiple sequence alignment with hierarchical clustering. Nucleic Acids Res 16:10881-10890.
