# Supplementary material for: Modulation of Quorum Sensing as an Adaptation to Nodule Cell Infection during Experimental Evolution of Legume Symbionts
Source: mBio. 2020 Jan 28;11(1):e03129-19. doi: 10.1128/mBio.03129-19 (PMC6989110; doi:10.1128/mBio.03129-19)
Supplement: FIG S2 [file mBio.03129-19-sf002.pdf]

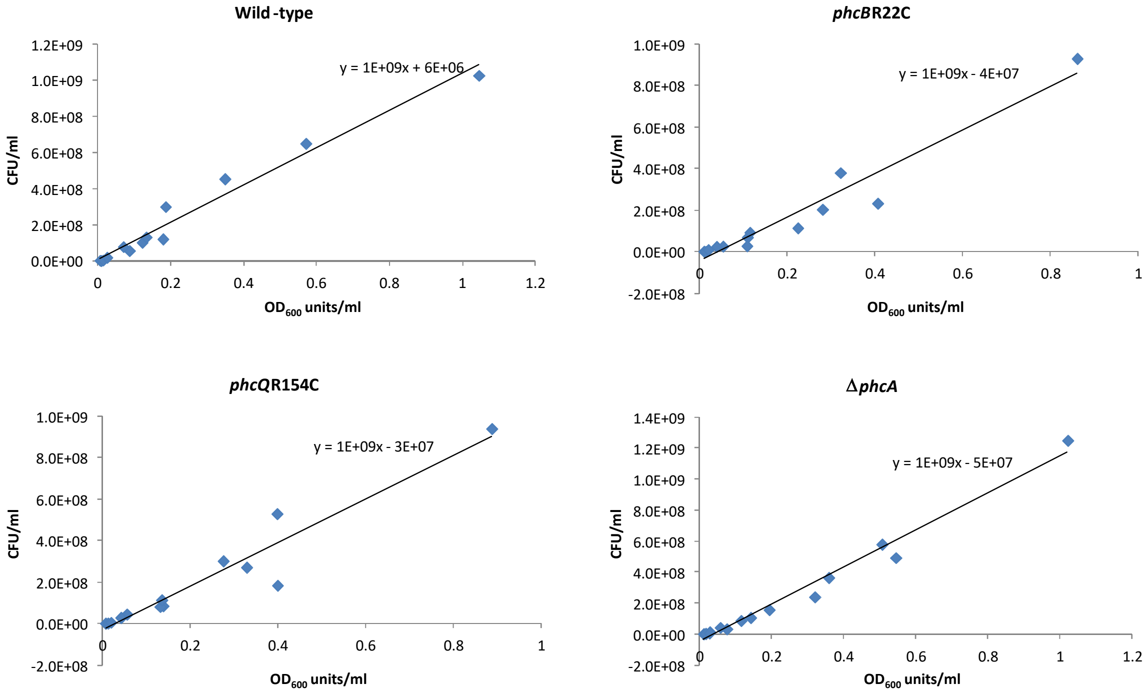

Figure S2. Correspondence between the OD_{600} units and the colony forming units (CFU) for the GMI1000pRalta *hrpG* strain (wild-type) and its derivative mutants.

Bacteria grown in rich BG medium were serially diluted and plated at different OD_{600} units/ml. Data are from three independent experiments. A linear regression model and the associated equation is indicated for each strain. The four strains have similar correspondences between OD_{600} units and CFU.
