# Supplementary material for: Modulation of Quorum Sensing as an Adaptation to Nodule Cell Infection during Experimental Evolution of Legume Symbionts
Source: mBio. 2020 Jan 28;11(1):e03129-19. doi: 10.1128/mBio.03129-19 (PMC6989110; doi:10.1128/mBio.03129-19)
Supplement: FIG S3 [file mBio.03129-19-sf003.pdf]

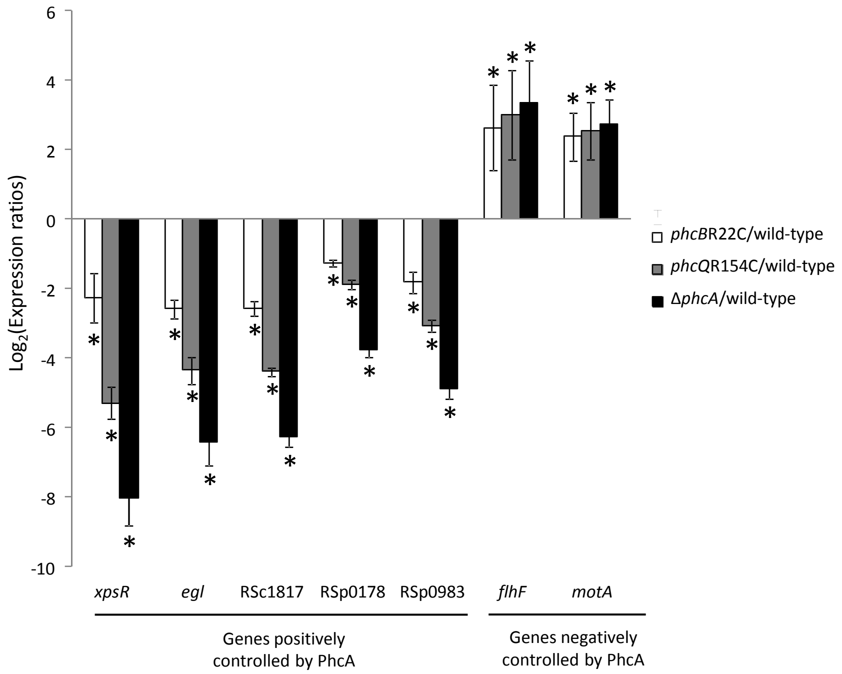

Figure S3 Expression of PhcA target genes in the GMI1000pRalta *hrpG* chimeric strain and the *phcB*R22C, *phcQ*R154C and Δ*phcA* derivative mutants. Strains were cultivated in BG medium until OD_{600} was around 0.1. Raw expression levels were normalized by *rplM* and *rpoA* expression and ratios of gene expression in mutants versus wild-type strain were calculated. Data were obtained from three independent experiments and are presented as mean Log_2(ratios)±standard deviations. Genes positively regulated by PhcA were repressed in *phcB*R22C, *phcQ*R154C as well as Δ*phcA* mutants. Genes negatively regulated by PhcA were found induced in *phcB*R22C, *phcQ*R154C as well as Δ*phcA* mutants.

* Significantly differentially expressed in the mutants compared to the wild-type strain (*P*<0.05, Student *t*-test).
