# Supplementary material for: Modulation of Quorum Sensing as an Adaptation to Nodule Cell Infection during Experimental Evolution of Legume Symbionts
Source: mBio. 2020 Jan 28;11(1):e03129-19. doi: 10.1128/mBio.03129-19 (PMC6989110; doi:10.1128/mBio.03129-19)
Supplement: FIG S4 [file mBio.03129-19-sf004.pdf]

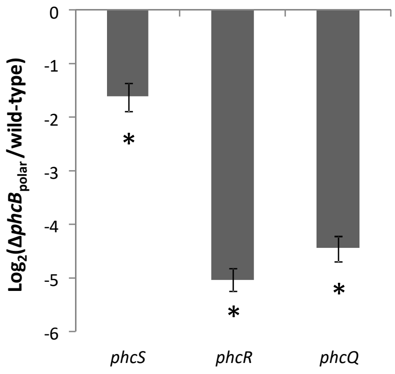

Figure S4. Expression of *phcS*, *phcR* and *phcQ* genes in the GMI1000pRalta *hrpG* chimeric strain and its *phcB* polar derivative mutant. Strains were cultivated in BG medium until mid-exponential phase. Raw expression levels were normalized by *rplM* expression and ratios of gene expression in mutants versus wild-type strain were calculated. Data were obtained from three independent experiments and are presented as Log_2(mean ratios)±standard deviations. * Significantly differentially expressed in the mutants compared to the wild-type strain (*P*<0.01, Student *t*-test).
