# Supplementary material for: Modulation of Quorum Sensing as an Adaptation to Nodule Cell Infection during Experimental Evolution of Legume Symbionts
Source: mBio. 2020 Jan 28;11(1):e03129-19. doi: 10.1128/mBio.03129-19 (PMC6989110; doi:10.1128/mBio.03129-19)
Supplement: TABLE S1 [file mBio.03129-19-st001.docx]

**Supplementary Table S1.** Strains and plasmids used in this study.

| **Organism  Strain ID** | **Common**  **name** | **Relevant characteristics** | **Reference/ source** |
| --- | --- | --- | --- |
| *E. coli* |  |  |  |
| DH5α | DH5α | *F recA lacZ*DM15 | Bethesda Research Laboratory |
| *R. solanacearum* |  |  |  |
| GMI1000 | GMI1000 | wild-type strain (phylotype IA) isolated from tomato in French Guyana | (1) |
| *C. taiwanensis* |  |  |  |
| LMG19424 |  | Wild-type strain isolated from *Mimosa pudica* in Taiwan | (2) |
| Chimeric *Ralstonia* | |  |  |
| CBM124 | GMI1000pRalta | GMI1000 pRalta::Tri, TriR | (3) |
| CBM125 | GMI1000pRalta *hrcV* | CBM124 *hrcV*::Ω, TriR, SpeR | (3) |
| CBM1627 | GMI1000pRalta *hrpG* | CBM124 *hrpG*, TriR | (4) |
| CBM212 | CBM212 | Nodulating ancestor mutated in *hrpG*, TriR, GenR | (3) |
| CBM349 | CBM349 | Nodulating ancestor mutated in *hrpG*, TriR, GenR | (3) |
| CBM356 | CBM356 | Nodulating ancestor mutated in *hrcV*, TriR, GenR | (3) |
| CBM2181 | E16 | Evolved clone from line E, cycle 16, TriR, GenR | (5) |
| CBM2184 | K16 | Evolved clone from line K, cycle 16, TriR, GenR | (5) |
| CBM1497 | M16 | Evolved clone from line M, cycle 16, TriR, GenR | (5) |
| CBM597 | M5 | Evolved clone from line M, cycle 5, TriR, GenR | (5) |
| RCM1865 | GMI1000pRalta *hrpG efpRE66K* | CBM1627 *efpR*E66K carrying a KanR cassette downstream *glmS,* TriR, KanR | (6) |
| RCM1878 | GMI1000pRalta *hrpG* GFP | CBM1627 carrying a P*psbA*-GFP fusion downstream *glmS,* TriR, KanR | This study |
| RCM1916 | GMI1000pRalta *hrpG xpsR* | CBM1627 carrying a spectinomycin resistance cassette into *xpsR,* TriR, SpeR | This study |
| RCM2288 | CBM212 *phcB*R22C | Evolved clone CBM212 *phcB*R22C carrying a P*psbA*-GFP fusion downstream *glmS*, TriR, GenR, KanR | (7) |
| RCM2289 | E16 *phcB*wt | Evolved clone E16 carrying the *phcB* wild-type allele and a P*psbA*-GFP fusion downstream *glmS*, TriR, GenR, KanR | (7) |
| RCM2290 | CBM356 *phcS*L161R | Evolved clone CBM356 *phcS*L161R carrying a P*psbA*-GFP fusion downstream *glmS*, TriR, GenR, KanR | (7) |
| RCM2291 | M5 *phcS*wt | Evolved clone M5 carrying the *phcS* wild-type allele and a P*psbA*-GFP fusion downstream *glmS*, TriR, GenR, KanR | (7) |
| RCM2292 | K16 *phcQ*wt | Evolved clone K16 carrying the *phcQ* wild-type allele and a P*psbA*-GFP fusion downstream *glmS*, TriR, GenR, KanR | (7) |
| RCM2346 | CBM349 *phcQ*R154C | Evolved clone CBM349 *phcQ*R154C carrying a P*psbA*-mCherry fusion downstream *glmS*, TriR, GenR, KanR | (7) |
| RCM2511 | GMI1000pRalta *hrpG phcB*R22C | CBM1627 *phcB*R22C carrying a P*psbA*-mCherry fusion downstream *glmS*, TriR, KanR | This study |
| RCM2495 | GMI1000pRalta *hrpG phcQ*R154C | CBM1627 *phcQ*R154C carrying a P*psbA*-mCherry fusion downstream *glmS*, TriR, KanR | This study |
| RCM2501 | GMI1000pRalta *hrpG phcS*L161R | CBM1627 *phcS*L161R carrying a P*psbA*-mCherry fusion downstream *glmS*, TriR, KanR | This study |
| RCM2801 | GMI1000pRalta *hrcV phcS*L161R | CBM125 *phcS*L161R carrying a P*psbA*-mCherry fusion downstream *glmS*, TriR, SpeR, KanR | This study |
| RCM2498 | GMI1000pRalta *hrpG* Δ*phcA* | CBM1627 Δ*phcA*, TriR, SpeR | This study |
| RCM2551 | GMI1000pRalta *hrpG* Δ*phcQ* | CBM1627 Δ*phcQ*, TriR | This study |
| RCM2565 | GMI1000pRalta *hrpG* Δ*phcB*_in frame_ | CBM1627 Δ*phcB* (in frame deletion), TriR | This study |
| RCM2499 | GMI1000pRalta *hrpG* Δ*phcB*_polar_ | CBM1627 Δ*phcB* (polar deletion), TriR, SpeR | This study |
| RCM2536 | GMI1000pRalta *hrpG efpR*E66K Δ*phcB*_polar_ | CBM1627 *efpR*E66K Δ*phcB* (polar deletion) carrying a P*psbA*-mCherry fusion downstream *glmS*, TriR, KanR, SpeR | This study |
| RCM2733 | GMI1000pRalta *hrpG phcB*R22C | CBM1627 *phcB*R22C carrying a SpeR cassette downstream *glmS*, TriR, SpeR | This study |
| RCM2734 | GMI1000pRalta *hrpG phcQ*R154C | CBM1627 *phcQ*R154C carrying a SpeR cassette downstream *glmS*, TriR, SpeR | This study |
| RCM2963 | GMI1000pRalta *hrpG efpR*E66K *phcB*R22C | CBM1627 *efpR*E66K *phcB*R22C carrying a KanR cassette and a P*psbA*-mCherry fusion downstream *glmS*, TriR, KanR | This study |
| RCM2964 | GMI1000pRalta *hrpG efpR*E66K *phcQ*R154C | CBM1627 *efpR*E66K *phcQ*R154C carrying a KanR cassette and a P*psbA*-mCherry fusion downstream *glmS*, TriR, KanR | This study |
| RCM2543 | GMI1000pRalta *phcB*R22C | CBM124 *phcB*R22C carrying a P*psbA*-GFP fusion downstream *glmS*, TriR, KanR | This study |
| RCM2544 | GMI1000pRalta *phcQ*R154C | CBM124 *phcQ*R154C carrying a P*psbA*-GFP fusion downstream *glmS*, TriR, KanR | This study |
| RCM2818 | GMI1000pRalta Δ*phcA* | CBM124 Δ*phcA*, TriR, SpeR | This study |
| RCM2966 | GMI1000pRalta Δ*phcB*_in frame_ | CBM124 Δ*phcB* (in frame deletion), TriR | This study |
| RCM2967 | GMI1000pRalta Δ*phcQ* | CBM124 Δ*phcQ*, TriR | This study |
| RCM2877 | GMI1000pRalta *hrpG* *phcQ*R154C Δ*phcB*_in frame_ | RCM2734 Δ*phcB* (in frame deletion) TriR, SpeR | This study |
| RCM2974 | GMI1000pRalta *hrpG* Δ*phcQ* Δ*phcB*_in frame_ | RCM2565 Δ*phcQ*, TriR | This study |
| **Plasmid names** | | **Relevant characteristics** | **Reference/ source** |
| pRalta | | Symbiotic plasmid of LMG19424 (0.5 Mb), likely auto-transferable* | (8) |
| pGEM-T | | Cloning vector | Promega |
| pEX18Tc | | Suicide plasmid carrying the *sacB* gene, TetR | (9) |
| pCBM142 | | pGEM-T plasmid carrying the SpeR cassette inserted in the intergenic region downstream *glmS*, SpeR, AmpR | (10) |
| pRCK-P*ps*-GFP | | Plasmid for *R. solanacearum* chromosomal integration of the constitutive *psbA* promoter fused to GFPuv into the intergenic region downstream *glmS*, KanR | (6) |
| pRCK-P*ps*-mCherry | | Plasmid for *R. solanacearum* chromosomal integration of the constitutive *psbA* promoter fused to mCherry into the intergenic region downstream *glmS*, KanR | (6) |
| pCZ388 | | pLAFR6 derivative containing a promotorless *lacZ* gene, GenR, TetR | (11) |
| pCBM181 (p*xpsR*-*lacZ*) | | pCZ388 containing the promoter region of *xpsR* (from 777 bp upstream the *xpsR* start codon to 33 pb downstream the *xpsR* start codon) fused to *lacZ*, GenR, TetR | This study |

* carries *tra* and *mob* genes. TriR, trimethoprim resistant. SpeR, spectinomycin resistant. GenR, gentamycin resistant. KanR, kanamycin resistant. AmpR, ampicillin resistant. TetR, tetracyclin resistant.

**References**

1. Boucher CA, Barberis PA, Trigalet AP, Demery DA. 1985. Transposon mutagenesis of *Pseudomonas solanacearum* isolation of Tn*5*-induced avirulent mutants. J Gen Microbiol 131:2449-2457.

2. Chen WM, Laevens S, Lee TM, Coenye T, De Vos P, Mergeay M, Vandamme P. 2001. *Ralstonia taiwanensis* sp nov., isolated from root nodules of *Mimosa* species and sputum of a cystic fibrosis patient. Int J Syst Evol Microbiol 51:1729-1735.

3. Marchetti M, Capela D, Glew M, Cruveiller S, Chane-Woon-Ming B, Gris C, Timmers T, Poinsot V, Gilbert LB, Heeb P, Medigue C, Batut J, Masson-Boivin C. 2010. Experimental evolution of a plant pathogen into a legume symbiont. PLoS Biol 8:e1000280.

4. Guan SH, Gris C, Cruveiller S, Pouzet C, Tasse L, Leru A, Maillard A, Médigue C, Batut J, Masson-Boivin C, Capela D. 2013. Experimental evolution of nodule intracellular infection in legume symbionts. ISME J 7:1367-77.

5. Marchetti M, Jauneau A, Capela D, Remigi P, Gris C, Batut J, Masson-Boivin C. 2014. Shaping bacterial symbiosis with legumes by experimental evolution. Mol Plant Microbe Interact 27:956-64.

6. Capela D, Marchetti M, Clérissi C, Perrier A, Guetta D, Gris C, Valls M, Jauneau A, Cruveiller S, Rocha EPC, Masson-Boivin C. 2017. Recruitment of a lineage-specific virulence regulatory pathway promotes intracellular infection by a plant pathogen experimentally evolved into a legume symbiont. Mol Biol Evol 34:2503-2521.

7. Clerissi C, Touchon M, Capela D, Tang M, Cruveiller S, Parker MA, Moulin L, Masson-Boivin C, Rocha EPC. 2018. Parallels between experimental and natural evolution of legume symbionts. Nat Commun 9:2264.

8. Amadou C, Pascal G, Mangenot S, Glew M, Bontemps C, Capela D, Carrere S, Cruveiller S, Dossat C, Lajus A, Marchetti M, Poinsot V, Rouy Z, Servin B, Saad M, Schenowitz C, Barbe V, Batut J, Medigue C, Masson-Boivin C. 2008. Genome sequence of the beta-rhizobium *Cupriavidus taiwanensis* and comparative genomics of rhizobia. Genome Res 18:1472-1483.

9. Hoang TT, Karkhoff-Schweizer RR, Kutchma AJ, Schweizer HP. 1998. A broad-host-range Flp-FRT recombination system for site-specific excision of chromosomally-located DNA sequences: application for isolation of unmarked *Pseudomonas aeruginosa* mutants. Gene 212:77-86.

10. Remigi P, Capela D, Clerissi C, Tasse L, Torchet R, Bouchez O, Batut J, Cruveiller S, Rocha EP, Masson-Boivin C. 2014. Transient hypermutagenesis accelerates the evolution of legume endosymbionts following horizontal gene transfer. PLoS Biol 12:e1001942.

11. Cunnac S, Boucher C, Genin S. 2004. Characterization of the cis-acting regulatory element controlling HrpB-mediated activation of the type III secretion system and effector genes in *Ralstonia solanacearum*. J Bacteriol 186:2309-2318.
